# Supplementary material for: Sex Steroid Hormone Analysis in Human Tear Fluid Using a Liquid Chromatography—Mass Spectrometry Method
Source: Int J Mol Sci. 2022 Nov 28;23(23):14864. doi: 10.3390/ijms232314864 (PMC9735929; doi:10.3390/ijms232314864)

## Supplementary material

*Brief Report*

**Sex steroid hormone analysis in human tear fluid using a liquid chromatography − mass spectrometry method**

Alexandra Robciuc[1,3], Hanna Savolainen-Peltonen[1], Mikko Haanpää[2], Jukka A. O. Moilanen[3] and Tomi Mikkola[1]

**Supplementary figure S1A: E1 chromatograms in blank samples.** Parent and transition ions $E_1$ [M−H]− m/z 269.1 to m/z 269.1 and m/z 145.0 in 0 pmol/L standard sample and water blank sample. No internal standard was added to the water blank.

| | Sample Name | Sample Type | Analyte Peak Name | Analyte Peak Area (counts) | Analyte Peak Height (cps) | Analyte Concent ration (pmol/l) | Analyte Retention Time (min) | Analyte Signal To Noise | Area Ratio | IS Peak Name | IS Peak Area (counts) | IS Peak Height (cps) | IS Retention Time (min) | Calculated Concentration (pmol/l) |
|---|---|---|---|---|---|---|---|---|---|---|---|---|---|---|
| 2 | St 0 pM | Standard | E1 | 1.64e+001 | 1.10e+002 | 0.00 | 7.61 | N/A | 0.00014 | E1-13C3 | 1.12e+005 | 3.65e+004 | 7.62 | 0.00000000000 |

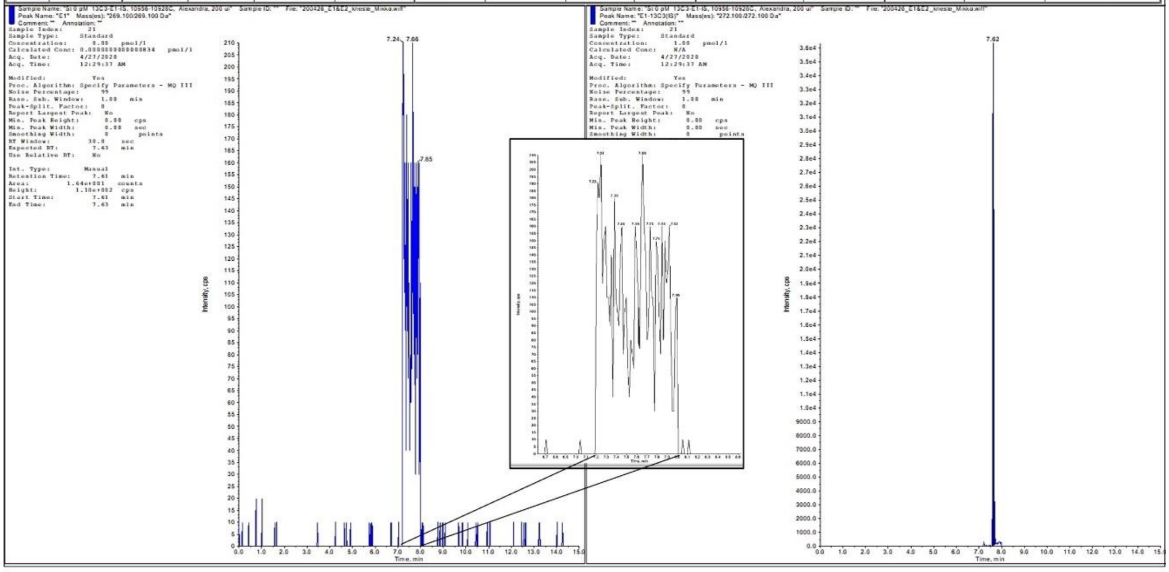

| | Sample Name | Sample Type | Analyte Peak Name | Analyte Peak Area (counts) | Analyte Peak Height (cps) | Analyte Concent ration (pmol/l) | Analyte Retention Time (min) | Analyte Signal To Noise | Area Ratio | IS Peak Name | IS Peak Area (counts) | IS Peak Height (cps) | IS Retention Time (min) | Calculated Concentration (pmol/l) |
|---|---|---|---|---|---|---|---|---|---|---|---|---|---|---|
| 1 | St 0 pM | Standard | E1 145.0 | 8.24e+002 | 4.78e+002 | 0.00 | 7.62 | N/A | 0.00097 | E1-13C3 148.0 | 8.42e+005 | 2.95e+005 | 7.63 | 0.00000000000 |

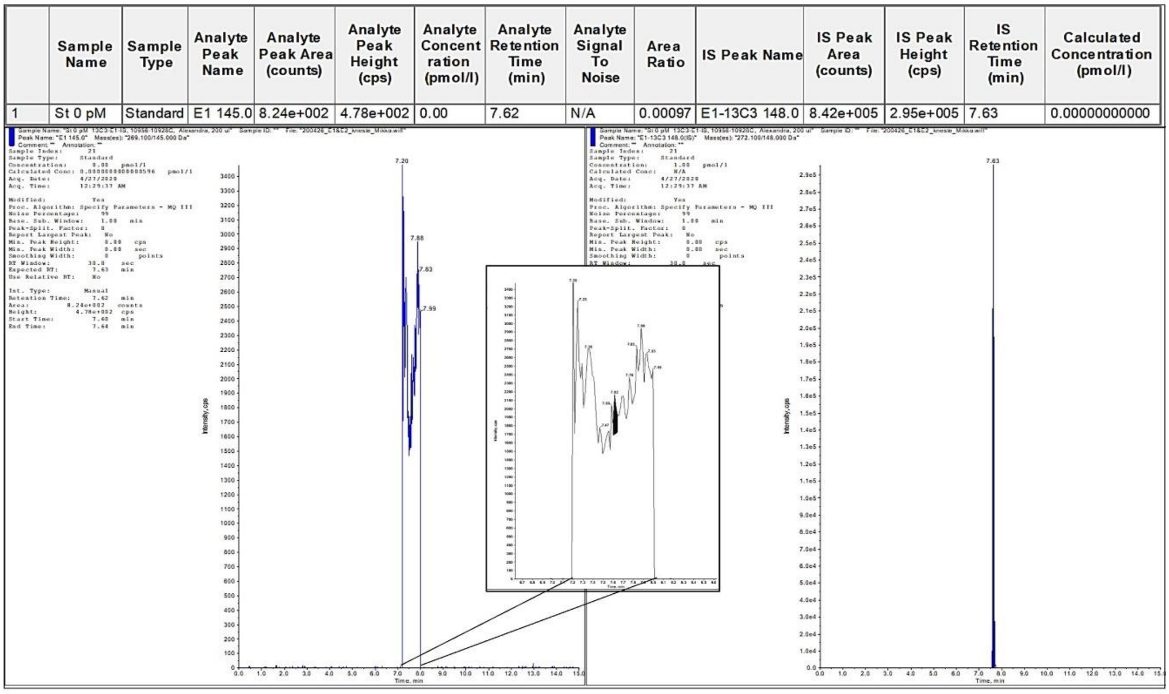

| | Sample Name | Sample Type | Analyte Peak Name | Analyte Peak Area (counts) | Analyte Peak Height (cps) | Analyte Concentration (pmol/l) | Analyte Retention Time (min) | Analyte Signal To Noise | Area Ratio | IS Peak Name | IS Peak Area (counts) | IS Peak Height (cps) | IS Retention Time (min) | Calculated Concentration (pmol/l) |
|---|---|---|---|---|---|---|---|---|---|---|---|---|---|---|
| 2 | St 0 pM | Standard | E1 | 1.64e+001 | 1.10e+002 | 0.00 | 7.61 | N/A | 0.00014 | E1-13C3 | 1.12e+005 | 3.65e+004 | 7.62 | 0.00000000000 |

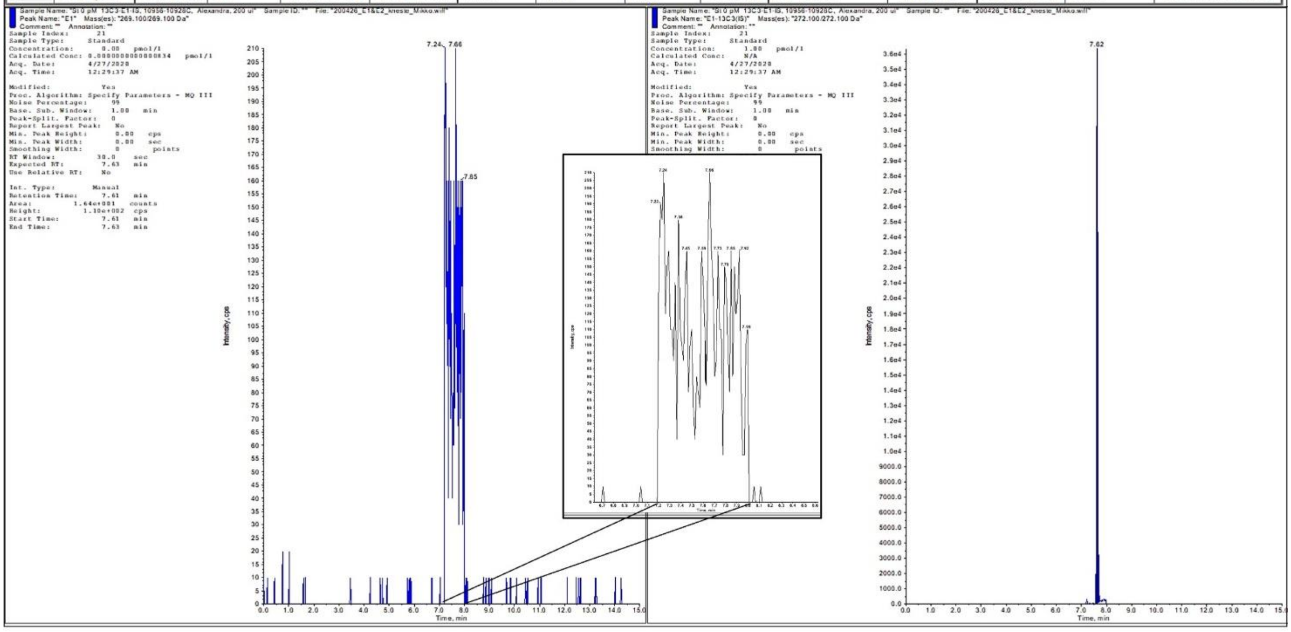

| | Sample Name | Sample Type | Analyte Peak Name | Analyte Peak Area (counts) | Analyte Peak Height (cps) | Analyte Concentration (pmol/l) | Analyte Retention Time (min) | Analyte Signal To Noise | Area Ratio | IS Peak Name | IS Peak Area (counts) | IS Peak Height (cps) | IS Retention Time (min) | Calculated Concentration (pmol/l) |
|---|---|---|---|---|---|---|---|---|---|---|---|---|---|---|
| 1 | St 0 pM | Standard | E1 145.0 | 8.24e+002 | 4.78e+002 | 0.00 | 7.62 | N/A | 0.00097 | E1-13C3 148.0 | 8.42e+005 | 2.95e+005 | 7.63 | 0.00000000000 |

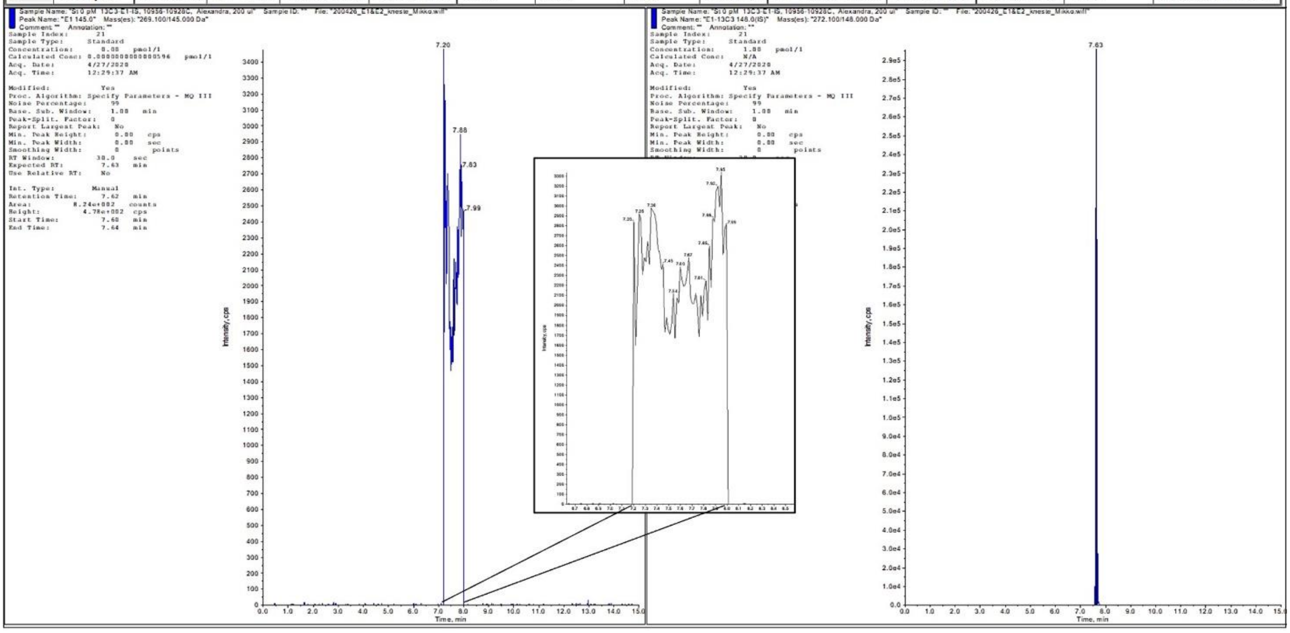

**Supplementary figure S1B: E2 chromatograms in blank samples.** Parent and transition ions $E_2$ [M−H]− m/z 271.2 to m/z 271.2 and m/z 183.1 in 0 pmol/L standard sample and water blank sample. No internal standard was added to the water blank.

| | Sample Name | Sample Type | Analyte Peak Name | Analyte Peak Area (counts) | Analyte Peak Height (cps) | Analyte Concentration (pmol/l) | Analyte Retention Time (min) | Analyte Signal To Noise | Area Ratio | IS Peak Name | IS Peak Area (counts) | IS Peak Height (cps) | IS Retention Time (min) | Calculated Concentration (pmol/l) |
|---|---|---|---|---|---|---|---|---|---|---|---|---|---|---|
| 2 | St 0 pM | Standard | E2 | 3.41e+002 | 1.49e+002 | 0.00 | 7.48 | N/A | 0.00037 | E2-13C3 | 9.00e+005 | 2.89e+005 | 7.46 | 0.00000000000 |

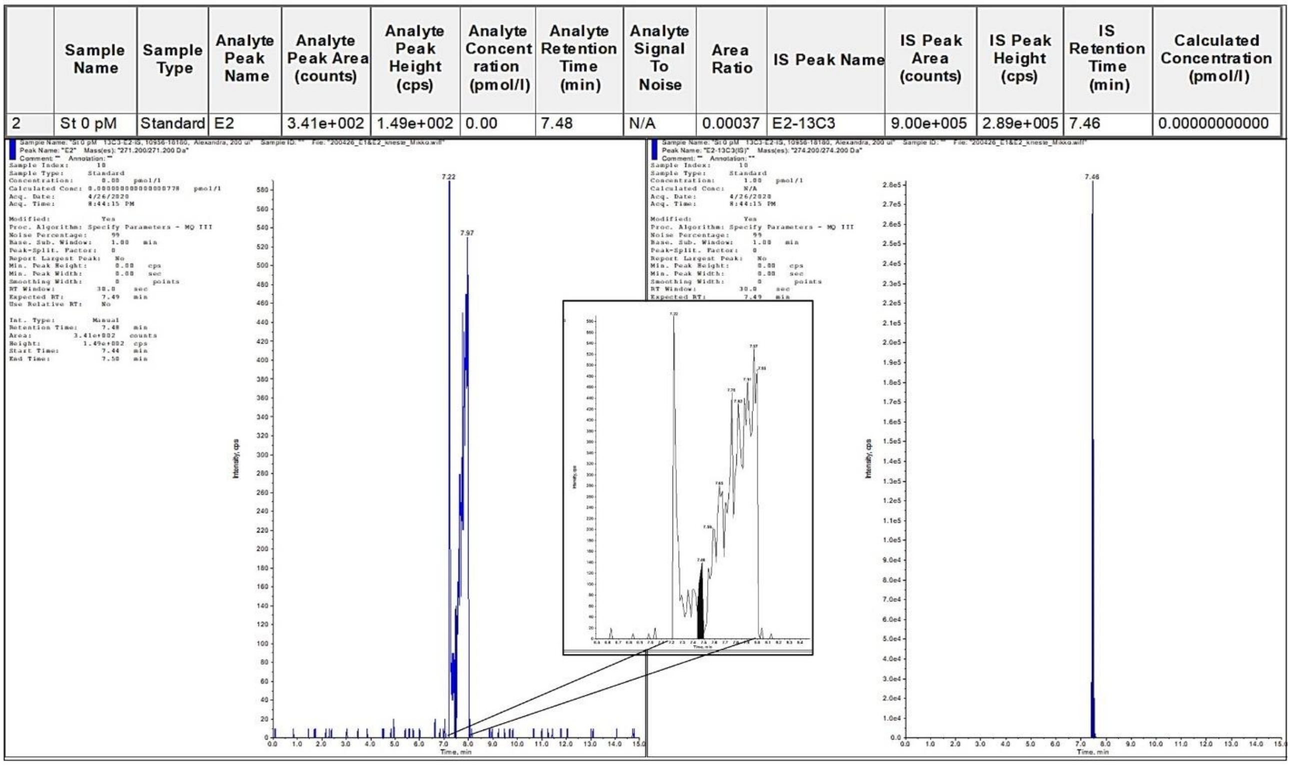

| | Sample Name | Sample Type | Analyte Peak Name | Analyte Peak Area (counts) | Analyte Peak Height (cps) | Analyte Concentration (pmol/l) | Analyte Retention Time (min) | Analyte Signal To Noise | Area Ratio | IS Peak Name | IS Peak Area (counts) | IS Peak Height (cps) | IS Retention Time (min) | Calculated Concentration (pmol/l) |
|---|---|---|---|---|---|---|---|---|---|---|---|---|---|---|
| 1 | St 0 pM | Standard | E2 183.1 | 8.77e+001 | 3.00e+002 | 0.00 | 7.48 | N/A | 0.00019 | E2-13C3 186.1 | 4.55e+005 | 1.48e+005 | 7.46 | < 0 |

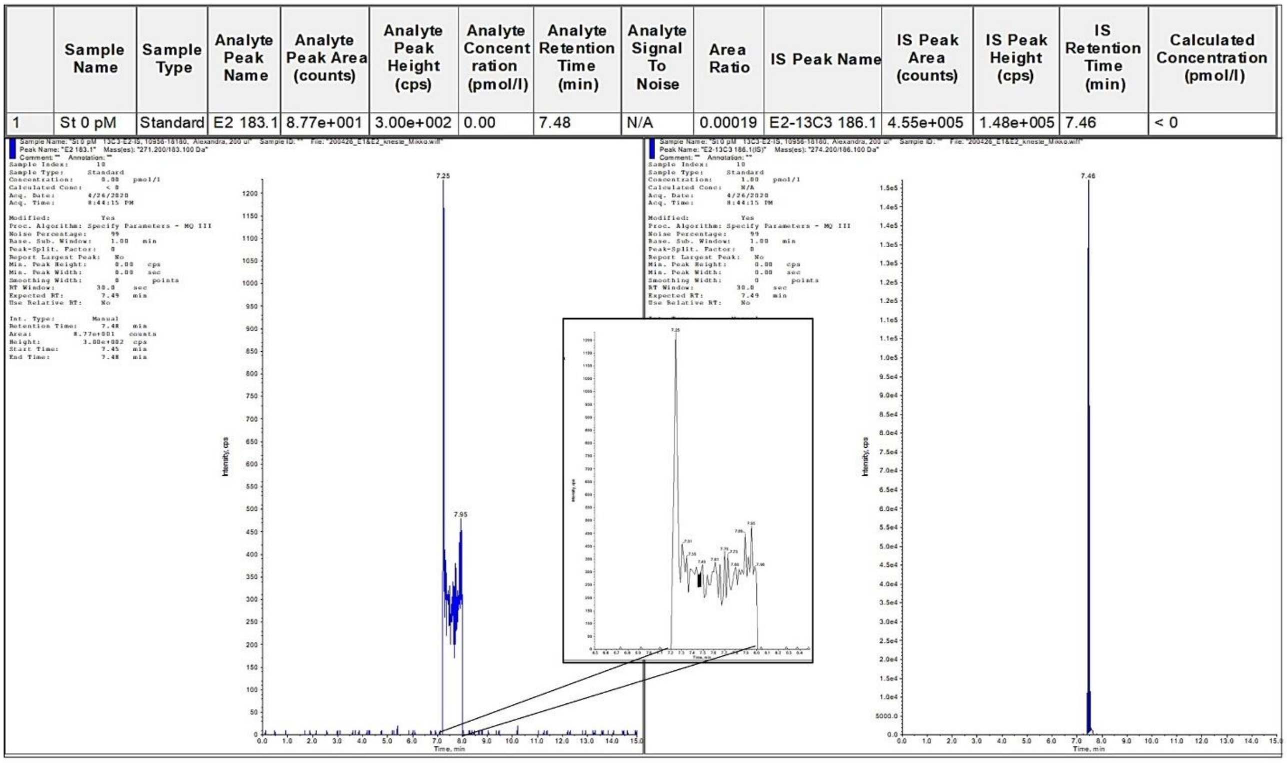

| | Sample Name | Sample Type | Analyte Peak Name | Analyte Peak Area (counts) | Analyte Peak Height (cps) | Analyte Concentration (pmol/l) | Analyte Retention Time (min) | Analyte Signal To Noise | Area Ratio | IS Peak Name | IS Peak Area (counts) | IS Peak Height (cps) | IS Retention Time (min) | Calculated Concentration (pmol/l) |
|---|---|---|---|---|---|---|---|---|---|---|---|---|---|---|
| 20 | 2 10956-2 | Unknown | E2 | 3.34e+001 | 1.20e+002 | N/A | 7.48 | N/A | -1.61 | E2-13C3 | -2.07e+001 | 8.00e+001 | 7.47 | < 0 |

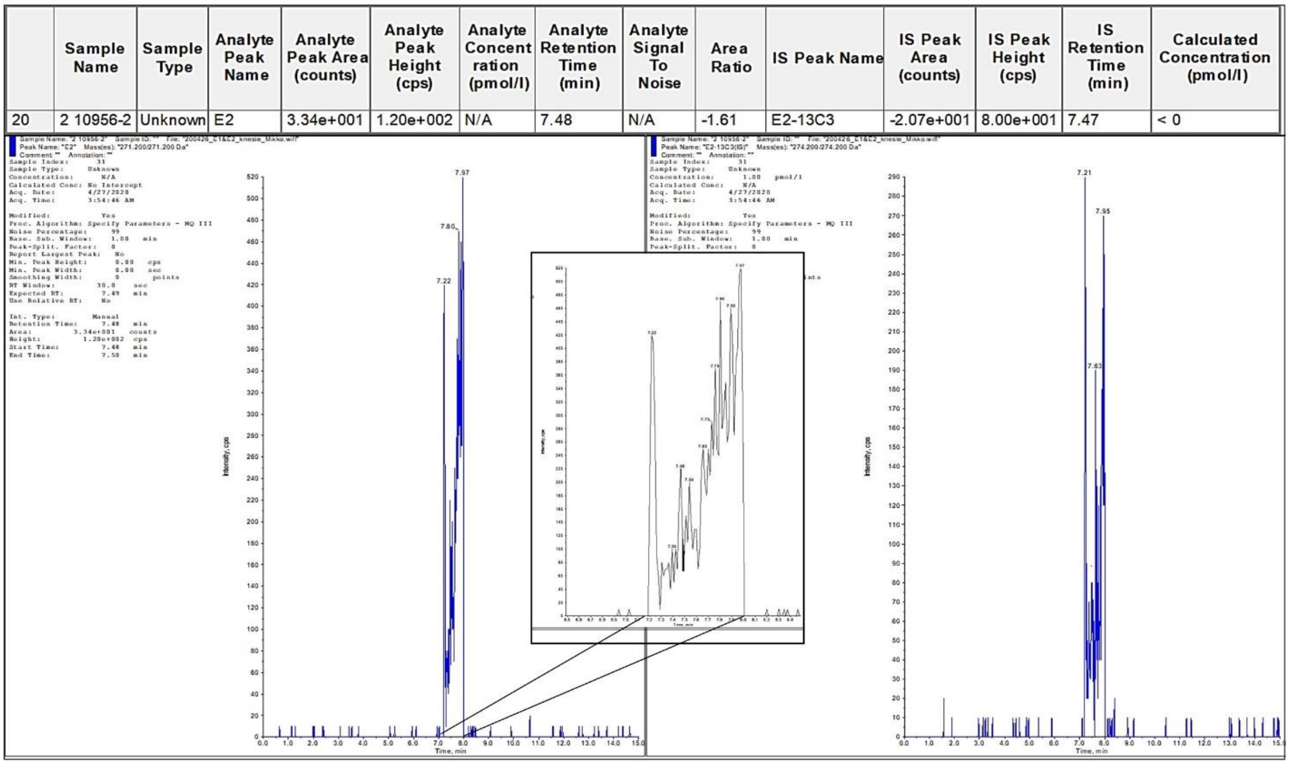


| | Sample Name | Sample Type | Analyte Peak Name | Analyte Peak Area (counts) | Analyte Peak Height (cps) | Analyte Concentration (pmol/l) | Analyte Retention Time (min) | Analyte Signal To Noise | Area Ratio | IS Peak Name | IS Peak Area (counts) | IS Peak Height (cps) | IS Retention Time (min) | Calculated Concentration (pmol/l) |
|---|---|---|---|---|---|---|---|---|---|---|---|---|---|---|
| 19 | 2 10956-2 | Unknown | E2 183.1 | 7.37e+001 | 4.70e+002 | N/A | 7.48 | N/A | -0.396 | E2-13C3 186.1 | -1.86e+002 | 1.50e+002 | 7.48 | < 0 |

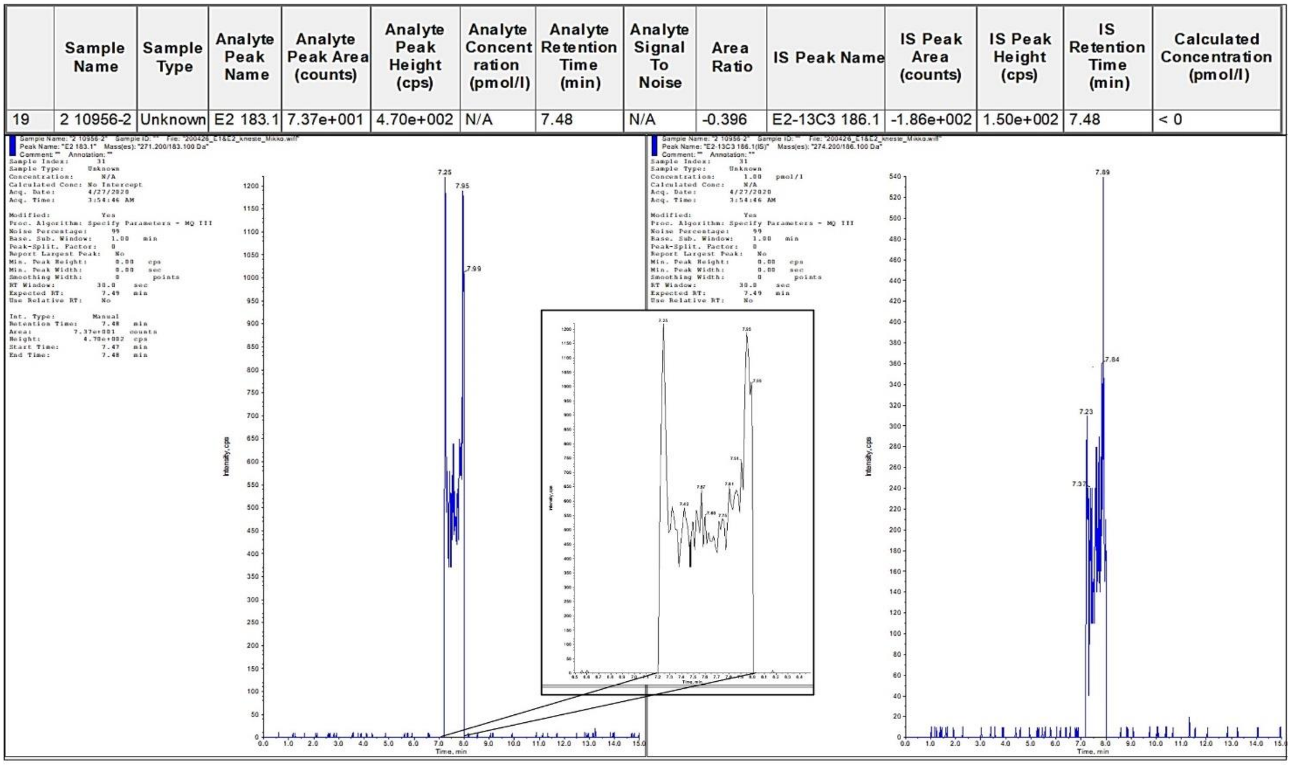


**Supplementary figure S1C: P4 chromatograms in blank samples.** Parent and transition ions P4 [M+H]+ m/z 315.1 to m/z 109.1 and m/z 96.9 in 0 pmol/L standard sample and water blank sample. No internal standard was added to the water blank.

| | Sample Name | Sample Type | Analyte Peak Name | Analyte Peak Area (counts) | Analyte Peak Height (cps) | Analyte Concentration (nmol/l) | Analyte Retention Time (min) | Analyte Signal To Noise | Area Ratio | IS Peak Name | IS Peak Area (counts) | IS Peak Height (cps) | IS Retention Time (min) | Calculated Concentration (nmol/l) |
|---|---|---|---|---|---|---|---|---|---|---|---|---|---|---|
| 1 | St 0 nM Prog | Standard | PROG 96.9 | 4.13e+002 | 2.82e+002 | 0.00 | 11.6 | N/A | 0.00006 | PROG-13C3 | 6.23e+006 | 2.01e+006 | 11.6 | 0.000000 |

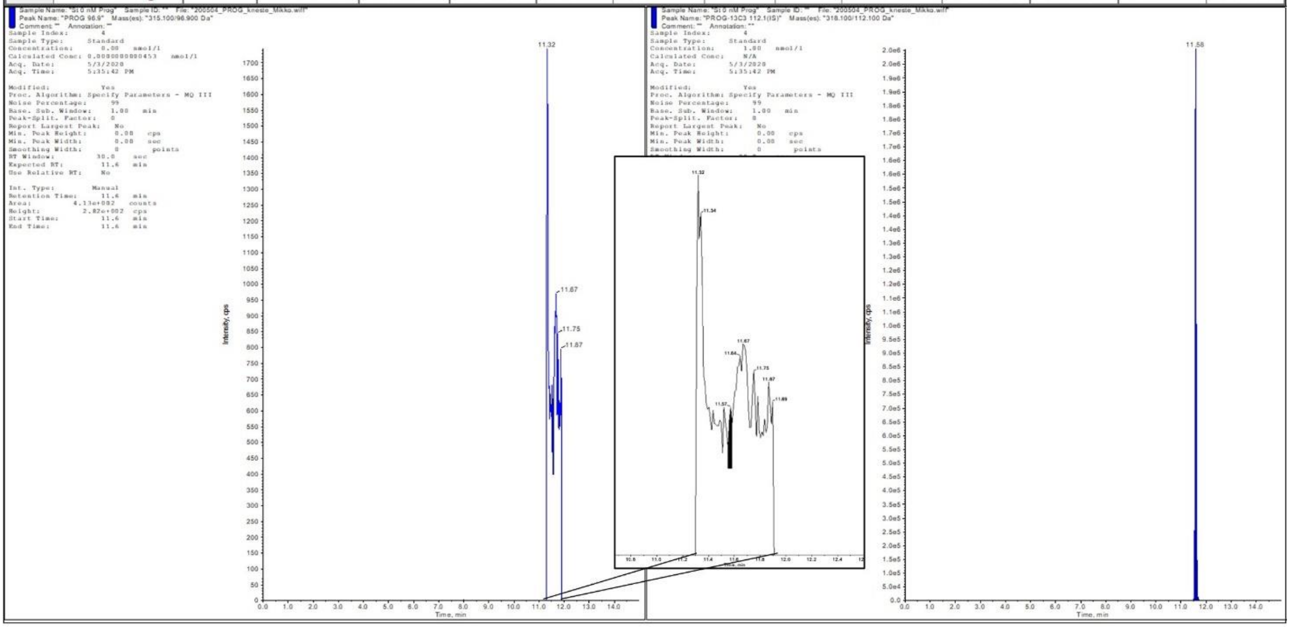

| | Sample Name | Sample Type | Analyte Peak Name | Analyte Peak Area (counts) | Analyte Peak Height (cps) | Analyte Concentration (nmol/l) | Analyte Retention Time (min) | Analyte Signal To Noise | Area Ratio | IS Peak Name | IS Peak Area (counts) | IS Peak Height (cps) | IS Retention Time (min) | Calculated Concentration (nmol/l) |
|---|---|---|---|---|---|---|---|---|---|---|---|---|---|---|
| 2 | St 0 nM Prog | Standard | PROG 109.1 | 7.31e+002 | 2.96e+002 | 0.00 | 11.6 | N/A | 0.00011 | PROG-13C3 | 6.23e+006 | 2.01e+006 | 11.6 | 0.000000 |

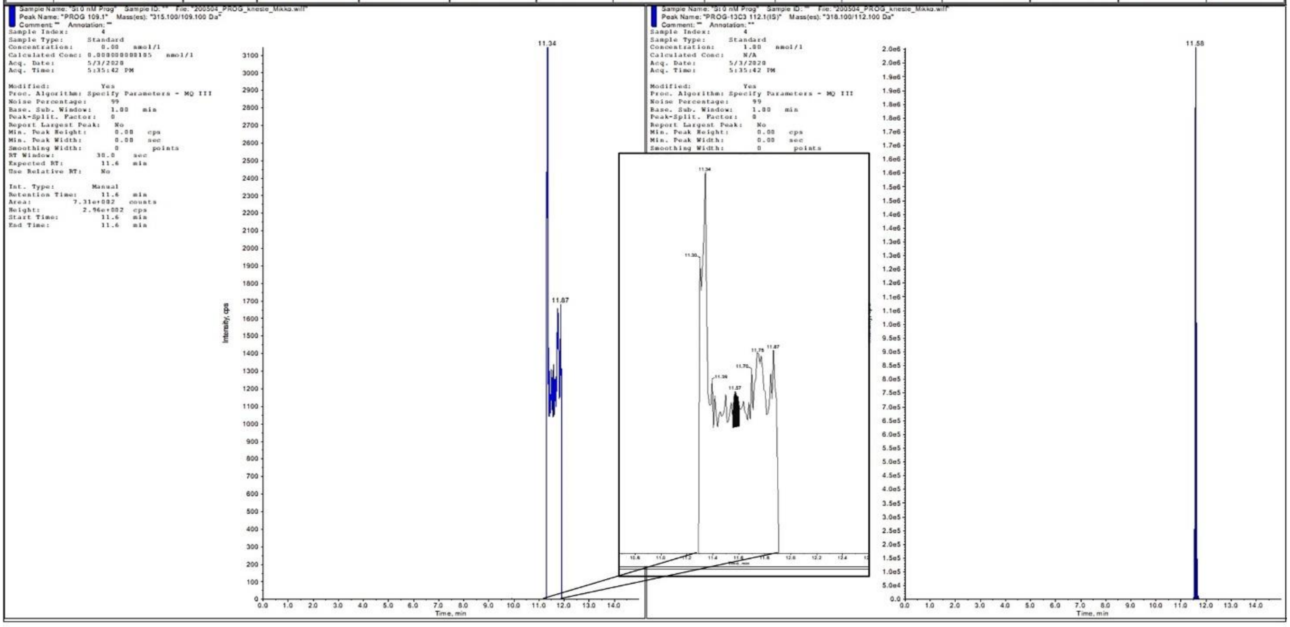

| | Sample Name | Sample Type | Analyte Peak Name | Analyte Peak Area (counts) | Analyte Peak Height (cps) | Analyte Concentration (nmol/l) | Analyte Retention Time (min) | Analyte Signal To Noise | Area Ratio | IS Peak Name | IS Peak Area (counts) | IS Peak Height (cps) | IS Retention Time (min) | Calculated Concentration (nmol/l) |
|---|---|---|---|---|---|---|---|---|---|---|---|---|---|---|
| 15 | 2 10956-2 | Unknown | PROG 96.9 | 1.06e+002 | 1.57e+002 | N/A | 11.6 | N/A | -0.590 | PROG-13C3 | -1.79e+00 | 1.42e+003 | 11.6 | < 0 |

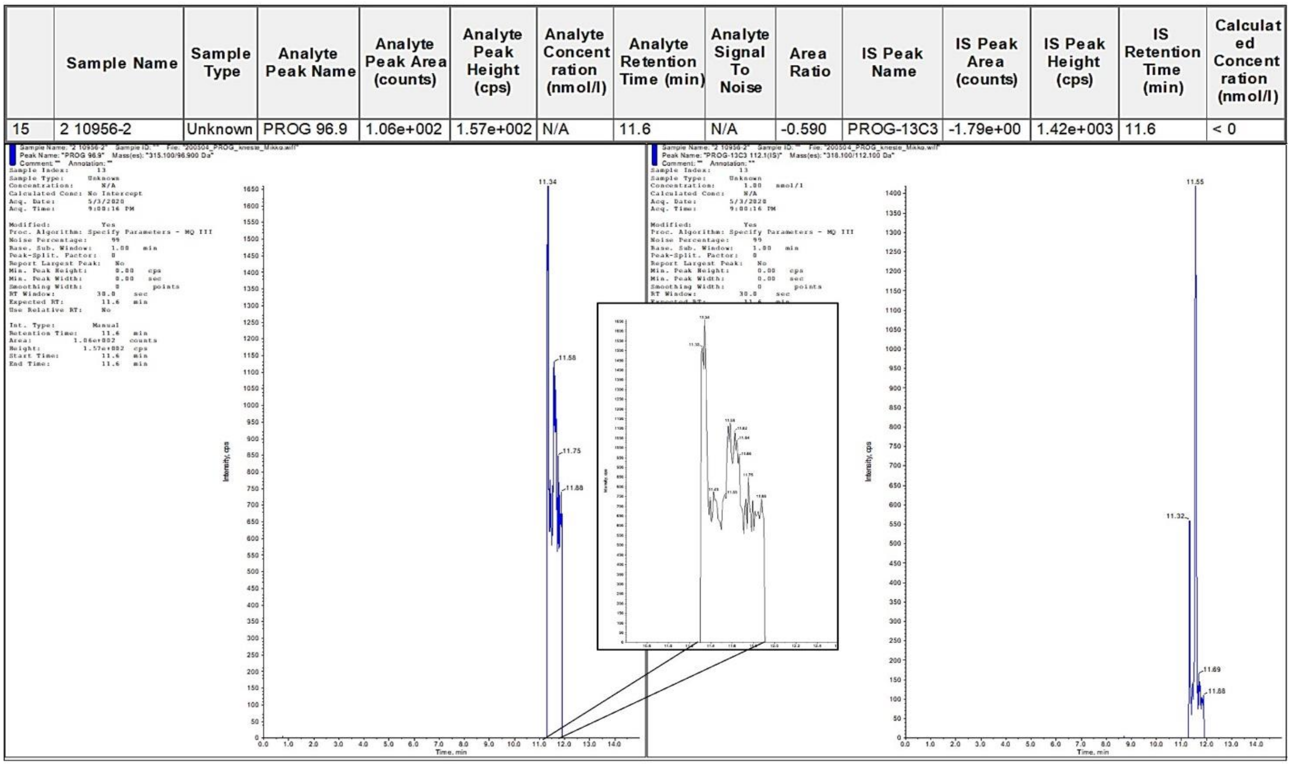

| | Sample Name | Sample Type | Analyte Peak Name | Analyte Peak Area (counts) | Analyte Peak Height (cps) | Analyte Concentration (nmol/l) | Analyte Retention Time (min) | Analyte Signal To Noise | Area Ratio | IS Peak Name | IS Peak Area (counts) | IS Peak Height (cps) | IS Retention Time (min) | Calculated Concentration (nmol/l) |
|---|---|---|---|---|---|---|---|---|---|---|---|---|---|---|
| 16 | 2 10956-2 | Unknown | PROG 109.1 | 3.04e+002 | 3.80e+002 | N/A | 11.6 | N/A | -1.70 | PROG-13C3 | -1.79e+00 | 1.42e+003 | 11.6 | < 0 |

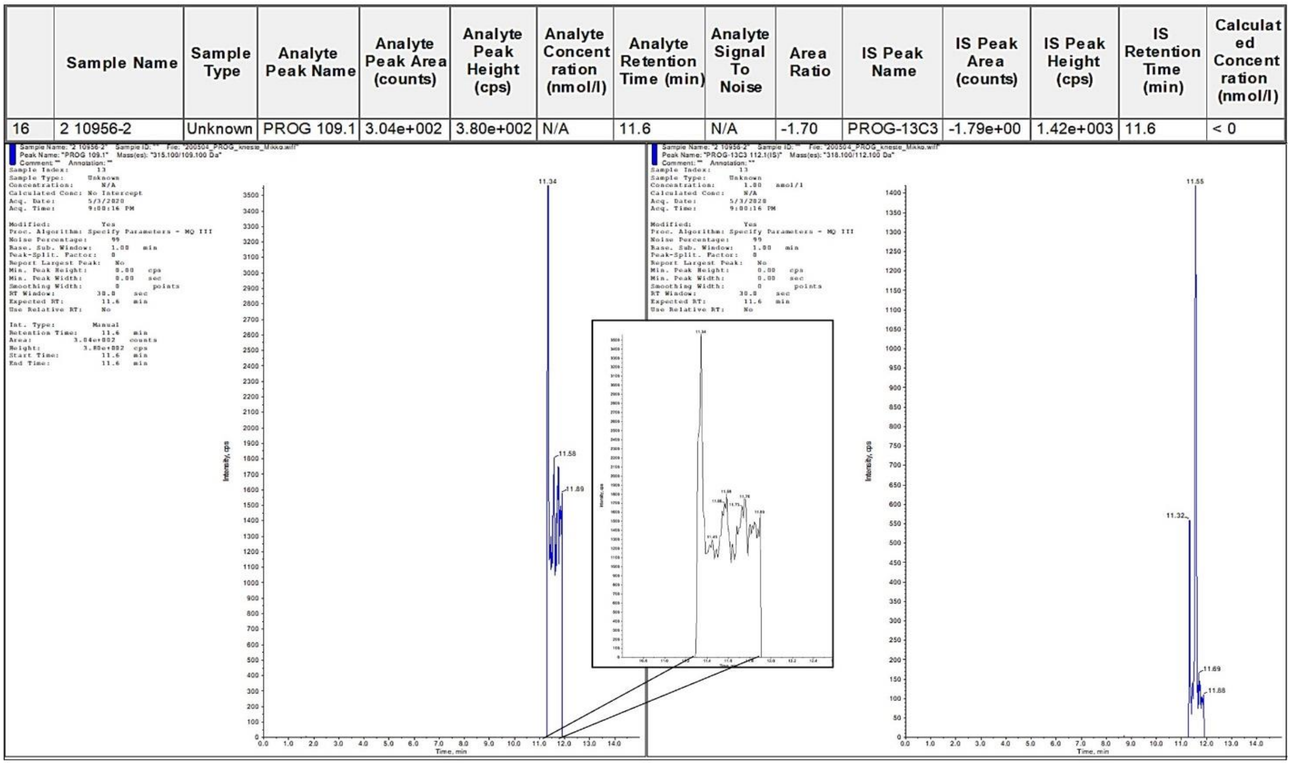

**Supplementary figure S1D: T4 chromatograms in blank samples.** Parent and transition ions T4 [M+H]+ m/z 289.1 to m/z 109.1 and m/z 97.0 in 0 pmol/L standard sample and water blank sample. No internal standard was added to the water blank.

| | Sample Name | Sample Type | Analyte Peak Name | Analyte Peak Area (counts) | Analyte Peak Height (cps) | Analyte Concentration (nmol/l) | Analyte Retention Time (min) | Analyte Signal To Noise | Area Ratio | IS Peak Name | IS Peak Area (counts) | IS Peak Height (cps) | IS Retention Time (min) | Calculated Concentration (nmol/l) |
|---|---|---|---|---|---|---|---|---|---|---|---|---|---|---|
| 1 | St 0 nM | Standard | TESTO 97.0 | 2.53e+002 | 2.18e+002 | 0.00 | 10.8 | N/A | 0.0000 | TESTO-13C3 100 | 2.64e+006 | 8.09e+005 | 10.8 | 0.00000000 |

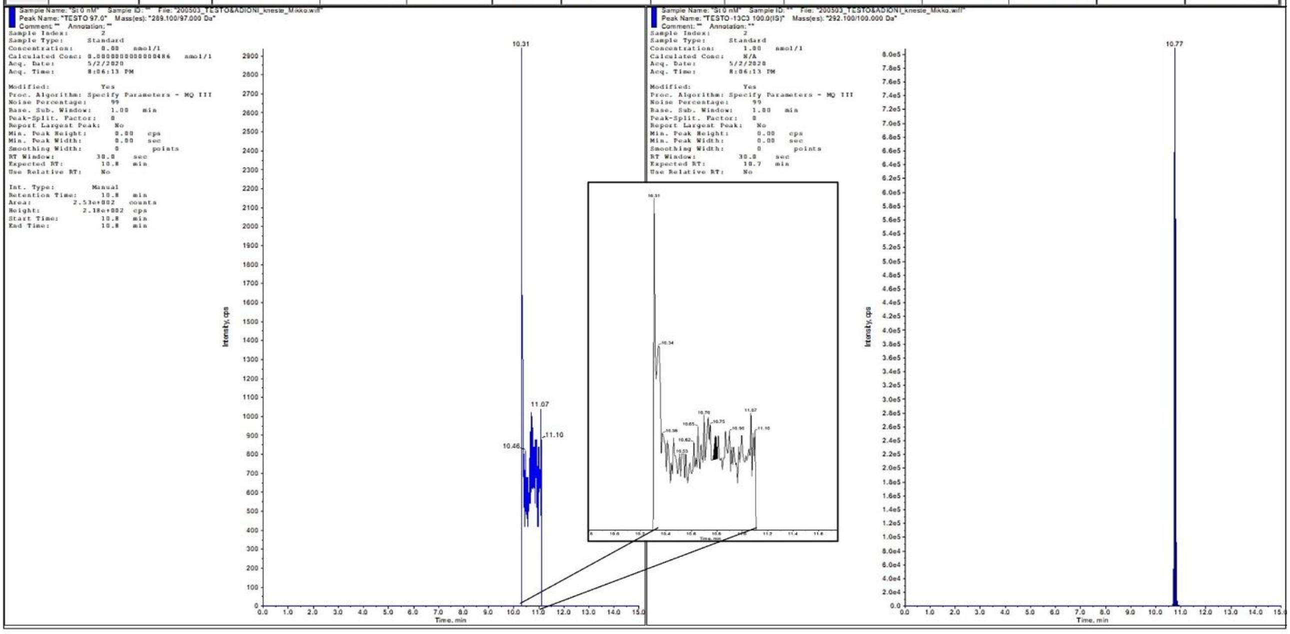

| | Sample Name | Sample Type | Analyte Peak Name | Analyte Peak Area (counts) | Analyte Peak Height (cps) | Analyte Concentration (nmol/l) | Analyte Retention Time (min) | Analyte Signal To Noise | Area Ratio | IS Peak Name | IS Peak Area (counts) | IS Peak Height (cps) | IS Retention Time (min) | Calculated Concentration (nmol/l) |
|---|---|---|---|---|---|---|---|---|---|---|---|---|---|---|
| 2 | St 0 nM | Standard | TESTO 109.1 | 2.42e+002 | 3.28e+002 | 0.00 | 10.8 | N/A | 0.0001 | TESTO-13C3 112 | 2.39e+006 | 7.66e+005 | 10.8 | 0.00000000 |

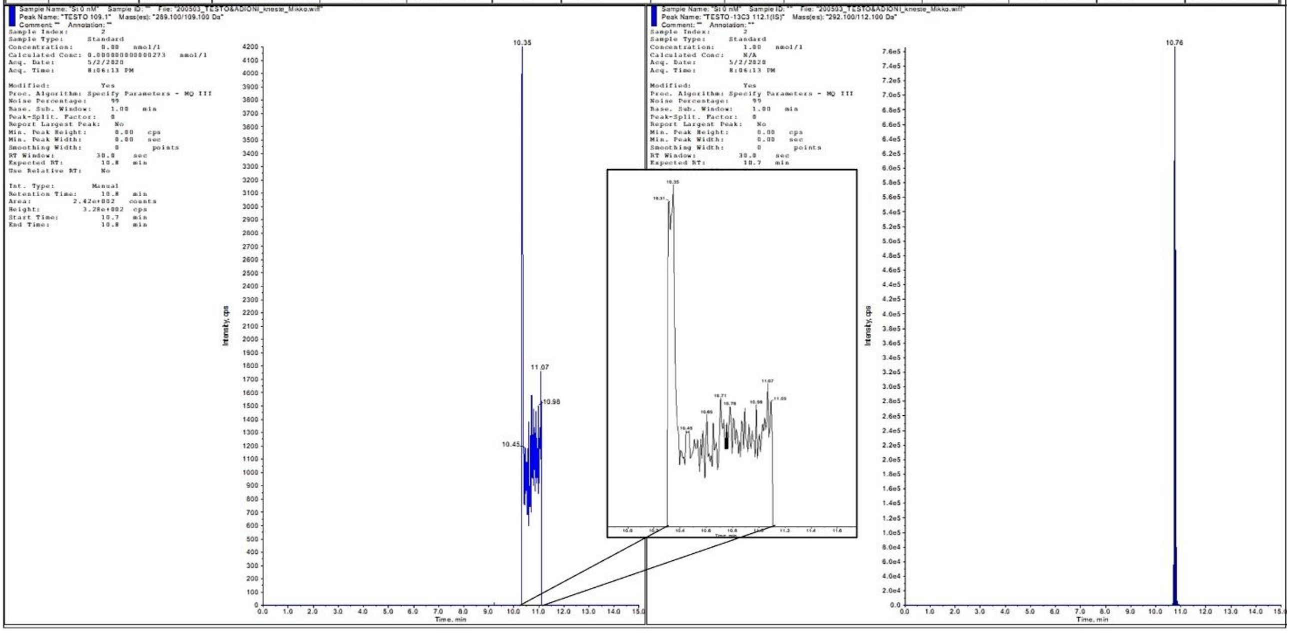

| | Sample Name | Sample Type | Analyte Peak Name | Analyte Peak Area (counts) | Analyte Peak Height (cps) | Analyte Concentration (nmol/l) | Analyte Retention Time (min) | Analyte Signal To Noise | Area Ratio | IS Peak Name | IS Peak Area (counts) | IS Peak Height (cps) | IS Retention Time (min) | Calculated Concentration (nmol/l) |
|---|---|---|---|---|---|---|---|---|---|---|---|---|---|---|
| 33 | 2 10956-2 | Unknown | TESTO 97.0 | 1.87e+002 | 1.98e+002 | N/A | 10.8 | N/A | -1.74 | TESTO-13C3 100. | -1.07e+00 | 6.60e+002 | 10.8 | < 0 |

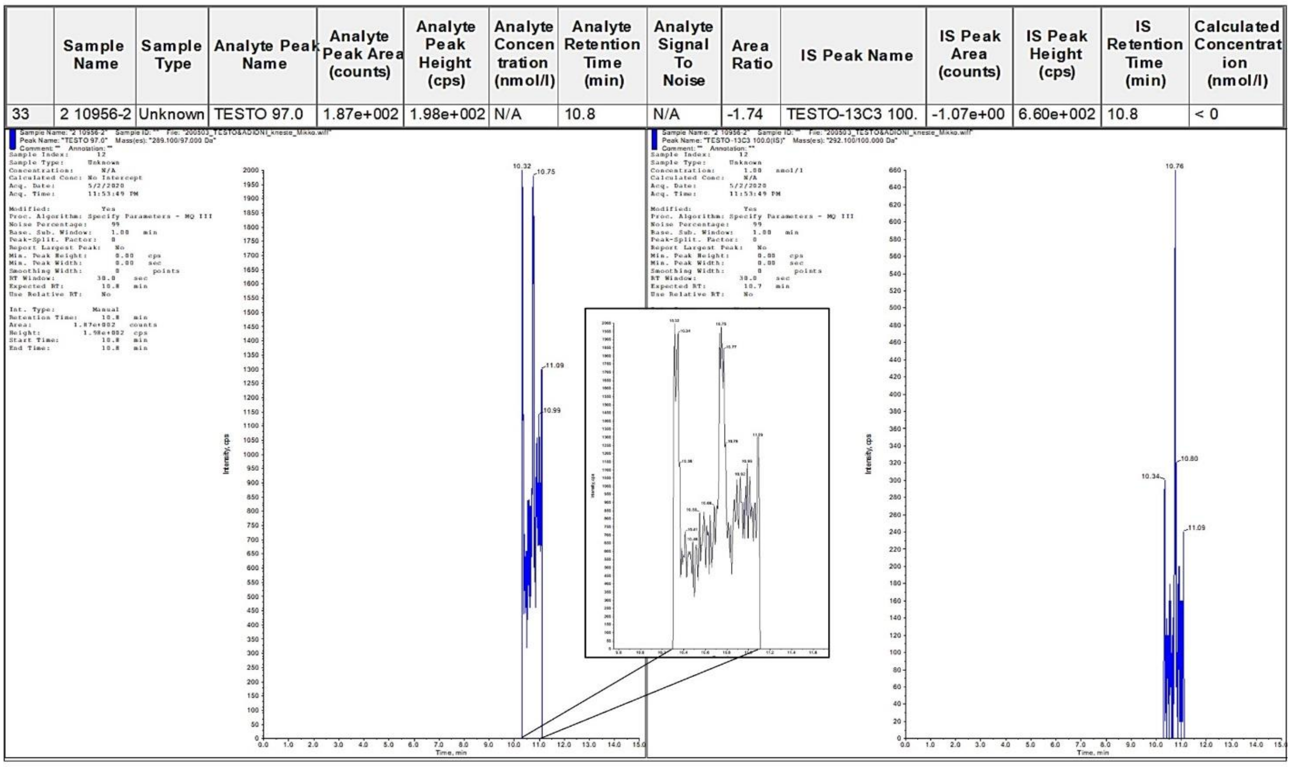

| | Sample Name | Sample Type | Analyte Peak Name | Analyte Peak Area (counts) | Analyte Peak Height (cps) | Analyte Concentration (nmol/l) | Analyte Retention Time (min) | Analyte Signal To Noise | Area Ratio | IS Peak Name | IS Peak Area (counts) | IS Peak Height (cps) | IS Retention Time (min) | Calculated Concentration (nmol/l) |
|---|---|---|---|---|---|---|---|---|---|---|---|---|---|---|
| 34 | 2 10956-2 | Unknown | TESTO 109.1 | 8.76e+002 | 8.24e+002 | N/A | 10.8 | N/A | -2.57 | TESTO-13C3 112. | -3.41e+00 | 0.00e+000 | 10.8 | < 0 |

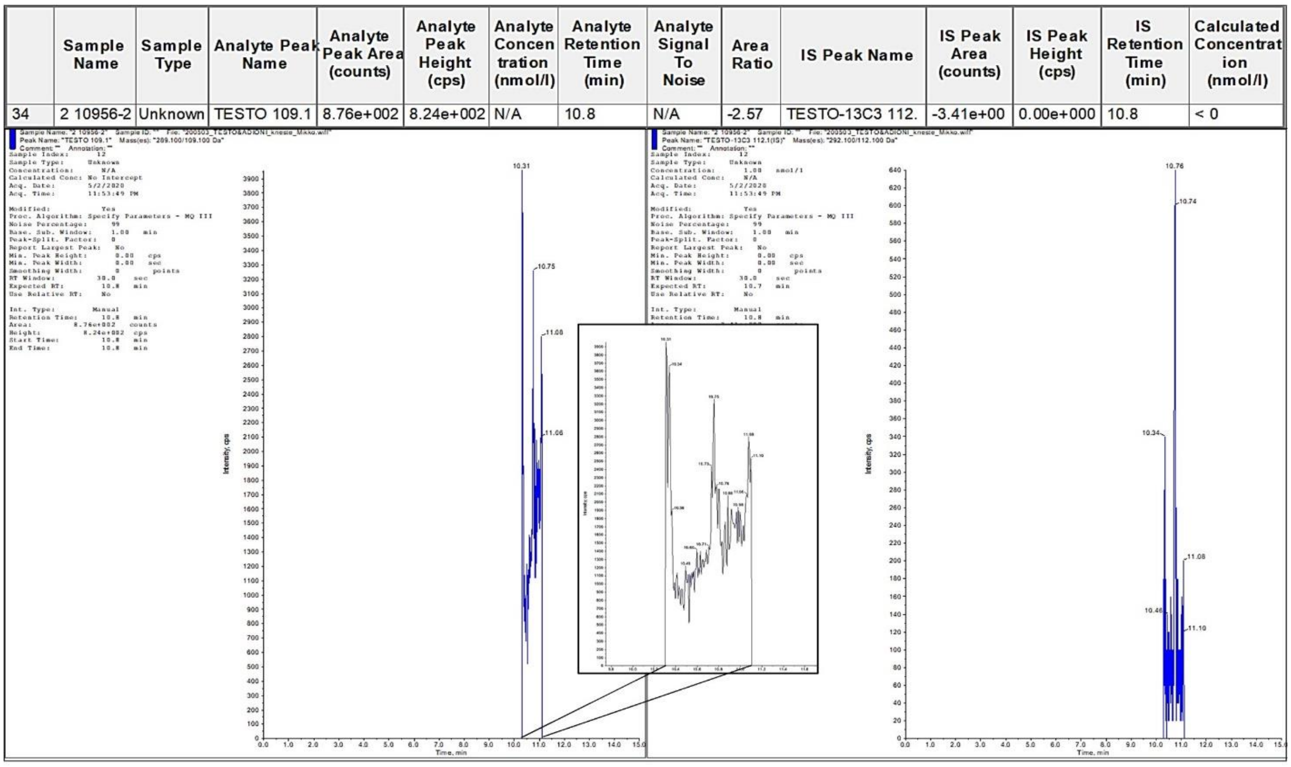

**Supplementary figure S1E: A4 chromatograms in blank samples.** Parent and transition ions A4 [M+H]+ m/z 287.1 to m/z 109.2 and m/z 97.2 in 0 pmol/L standard sample and water blank sample. No internal standard was added to the water blank.

| | Sample Name | Sample Type | Analyte Peak Name | Analyte Peak Area (counts) | Analyte Peak Height (cps) | Analyte Concentration (nmol/l) | Analyte Retention Time (min) | Analyte Signal To Noise | Area Ratio | IS Peak Name | IS Peak Area (counts) | IS Peak Height (cps) | IS Retention Time (min) | Calculated Concentration (nmol/l) |
|---|---|---|---|---|---|---|---|---|---|---|---|---|---|---|
| 4 | St 0 nM | Standard | ADIONI 109. | 5.21e+002 | 5.13e+002 | 0.00 | 10.7 | N/A | 0.0002 | ADIONI-13C3 11 | 1.85e+006 | 5.90e+005 | 10.7 | 0.00000000 |

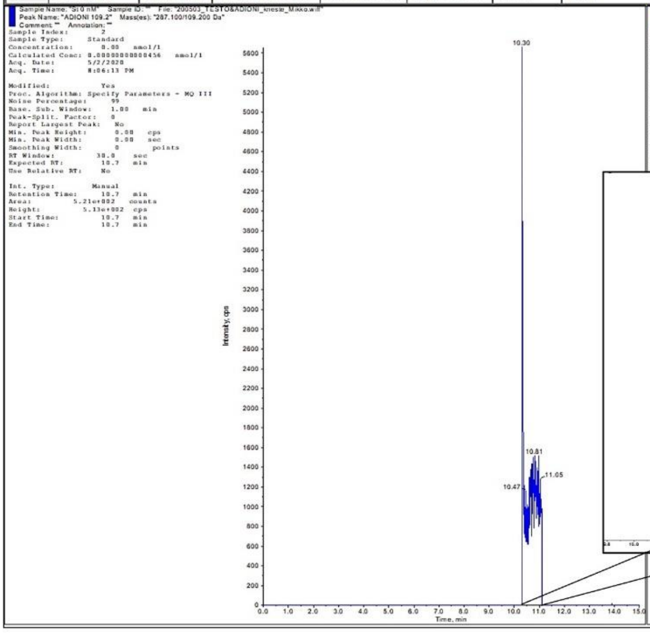


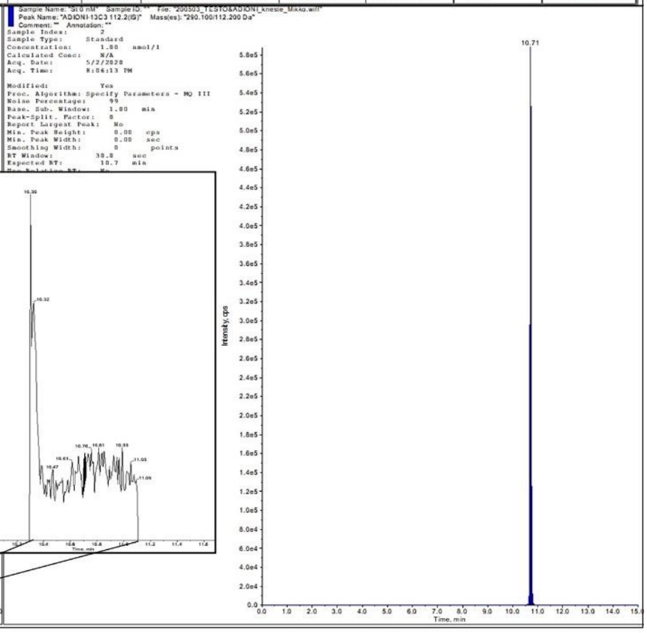


| | Sample Name | Sample Type | Analyte Peak Name | Analyte Peak Area (counts) | Analyte Peak Height (cps) | Analyte Concentration (nmol/l) | Analyte Retention Time (min) | Analyte Signal To Noise | Area Ratio | IS Peak Name | IS Peak Area (counts) | IS Peak Height (cps) | IS Retention Time (min) | Calculated Concentration (nmol/l) |
|---|---|---|---|---|---|---|---|---|---|---|---|---|---|---|
| 3 | St 0 nM | Standard | ADIONI 97.2 | 3.15e+003 | 1.90e+003 | 0.00 | 10.7 | N/A | 0.0012 | ADIONI-13C3 10 | 2.52e+006 | 7.81e+005 | 10.7 | 0.00000000 |

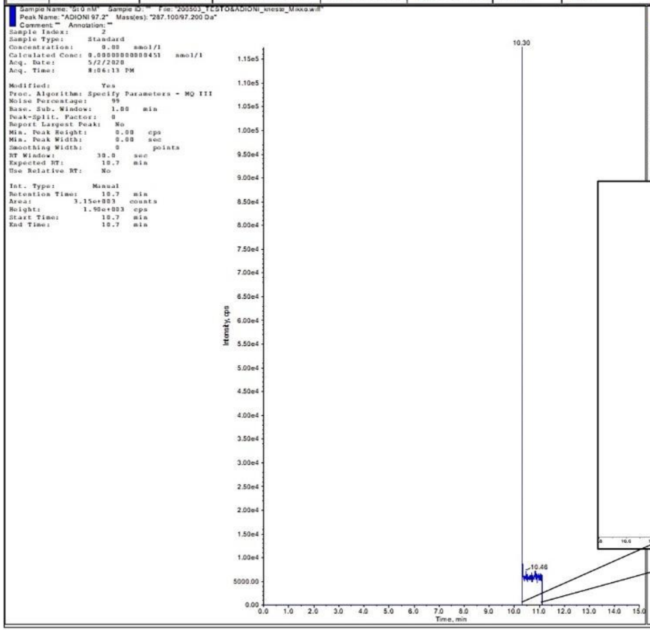


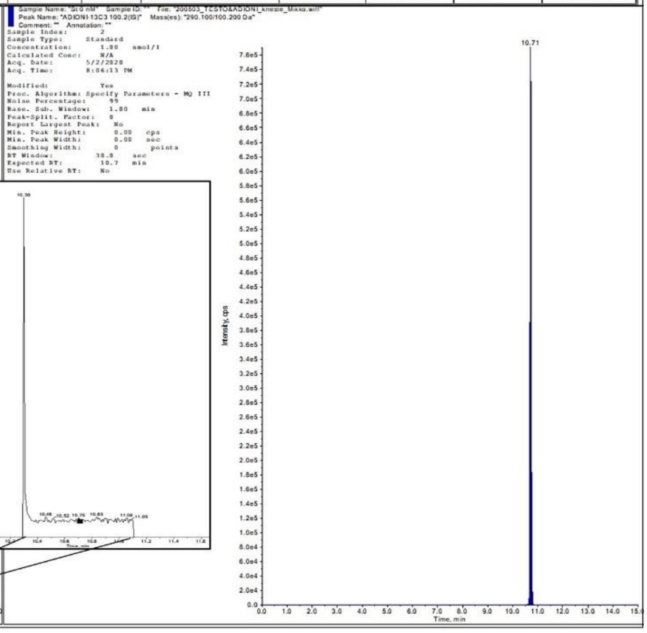

|  | Sample Name | Sample Type | Analyte Peak Name | Analyte Peak Area (counts) | Analyte Peak Height (cps) | Analyte Concentration (nmol/l) | Analyte Retention Time (min) | Analyte Signal To Noise | Area Ratio | IS Peak Name | IS Peak Area (counts) | IS Peak Height (cps) | IS Retention Time (min) | Calculated Concentration (nmol/l) |
|---|---|---|---|---|---|---|---|---|---|---|---|---|---|---|
| 36 | 2 10956-2 | Unknown | ADIONI 109. | 4.71e+002 | 5.26e+002 | N/A | 10.7 | N/A | -1.42 | ADIONI-13C3 112. | -3.31e+00 | 0.00e+000 | 10.7 | < 0 |

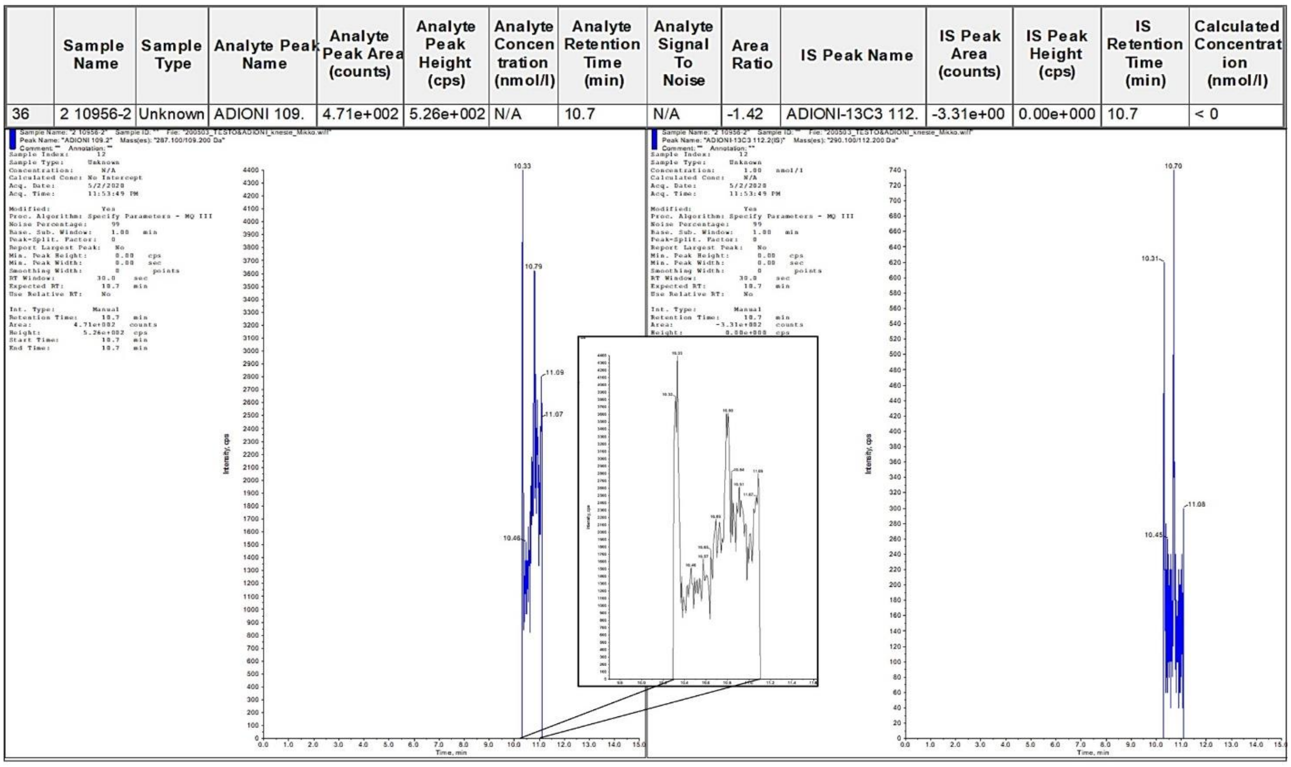

|  | Sample Name | Sample Type | Analyte Peak Name | Analyte Peak Area (counts) | Analyte Peak Height (cps) | Analyte Concentration (nmol/l) | Analyte Retention Time (min) | Analyte Signal To Noise | Area Ratio | IS Peak Name | IS Peak Area (counts) | IS Peak Height (cps) | IS Retention Time (min) | Calculated Concentration (nmol/l) |
|---|---|---|---|---|---|---|---|---|---|---|---|---|---|---|
| 35 | 2 10956-2 | Unknown | ADIONI 97.2 | 1.03e+003 | 1.24e+003 | N/A | 10.7 | N/A | -6.40 | ADIONI-13C3 100. | -1.60e+00 | 7.00e+002 | 10.7 | < 0 |

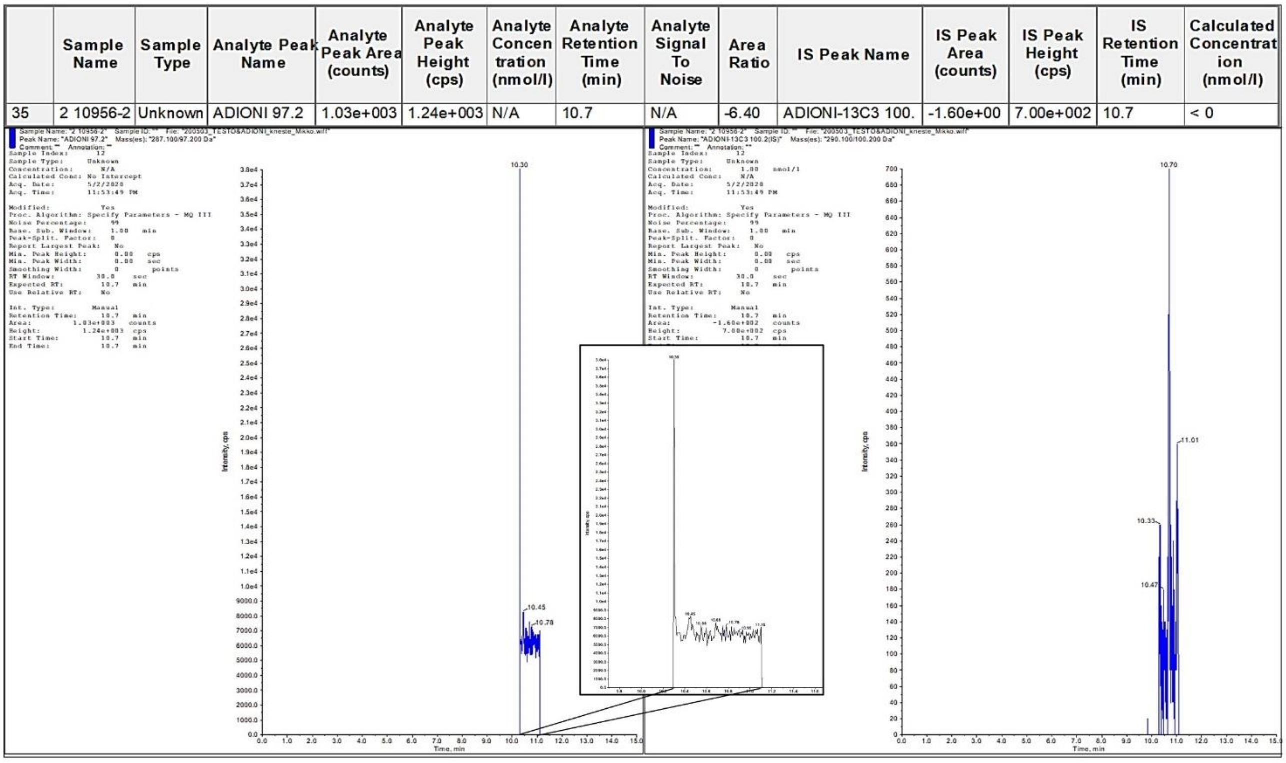

**Supplementary figure S1F: DHEA chromatograms in blank samples.** Parent and transition ions [M+NH4]+ m/z 306.1 to m/z 253.1 and m/z 213.3 in 0 pmol/L standard sample and water blank sample. No internal standard was added to the water blank.

| | Sample Name | Sample Type | Analyte Peak Name | Analyte Peak Area (counts) | Analyte Peak Height (cps) | Analyte Concentration (nmol/l) | Analyte Retention Time (min) | Analyte Signal To Noise | Area Ratio | IS Peak Name | IS Peak Area (counts) | IS Peak Height (cps) | IS Retention Time (min) | Calculated Concentration (nmol/l) |
|---|---|---|---|---|---|---|---|---|---|---|---|---|---|---|
| 5 | St 0 nM DHEA | Standard | DHEA-NH4 253.1 | 6.33e+002 | 5.14e+002 | 0.00 | 6.85 | N/A | 0.0005 | DHEA- | 1.16e+006 | 4.45e+005 | 6.85 | 0.00000000 |

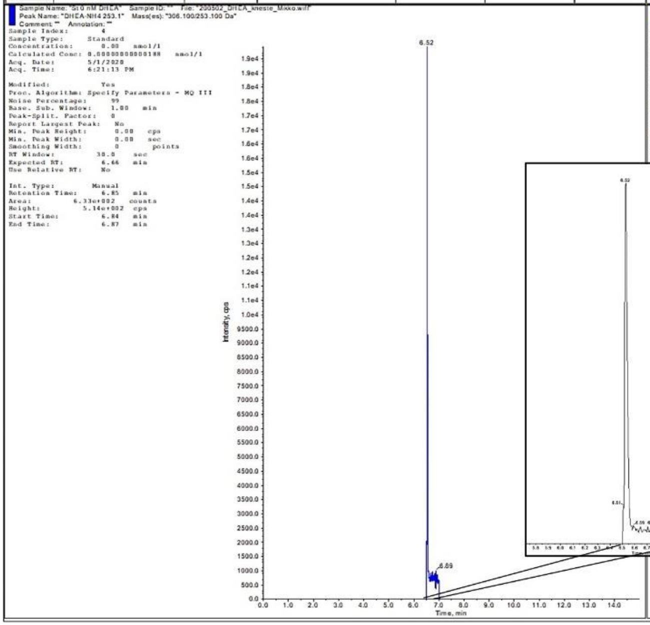

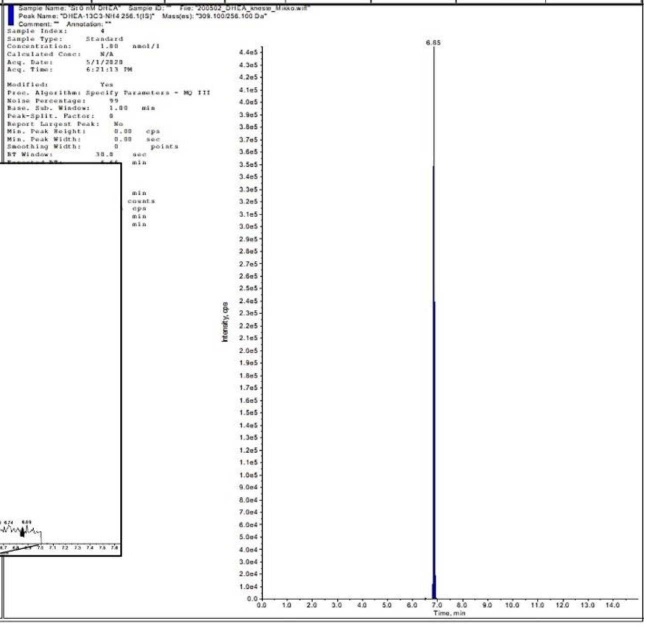

| | Sample Name | Sample Type | Analyte Peak Name | Analyte Peak Area (counts) | Analyte Peak Height (cps) | Analyte Concentration (nmol/l) | Analyte Retention Time (min) | Analyte Signal To Noise | Area Ratio | IS Peak Name | IS Peak Area (counts) | IS Peak Height (cps) | IS Retention Time (min) | Calculated Concentration (nmol/l) |
|---|---|---|---|---|---|---|---|---|---|---|---|---|---|---|
| 6 | St 0 nM DHEA | Standard | DHEA-NH4 213.3 | 4.45e+002 | 2.83e+002 | 0.00 | 6.83 | N/A | 0.0032 | DHEA- | 1.37e+005 | 5.19e+004 | 6.85 | 0.00000000 |

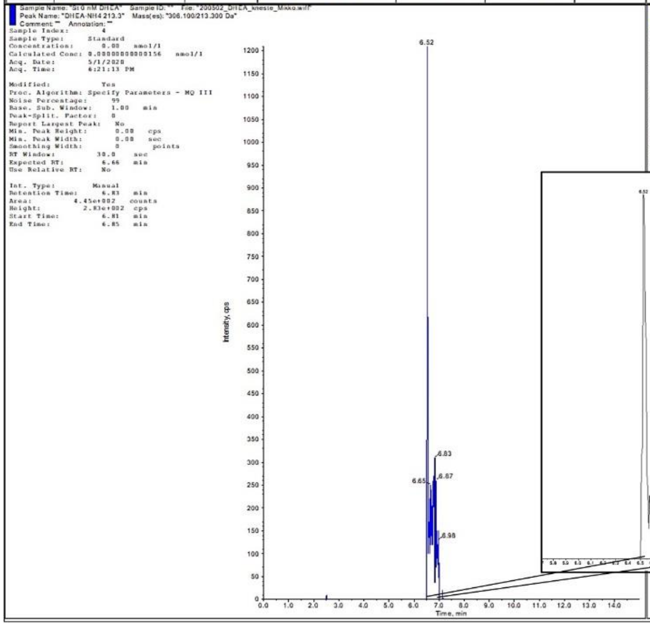

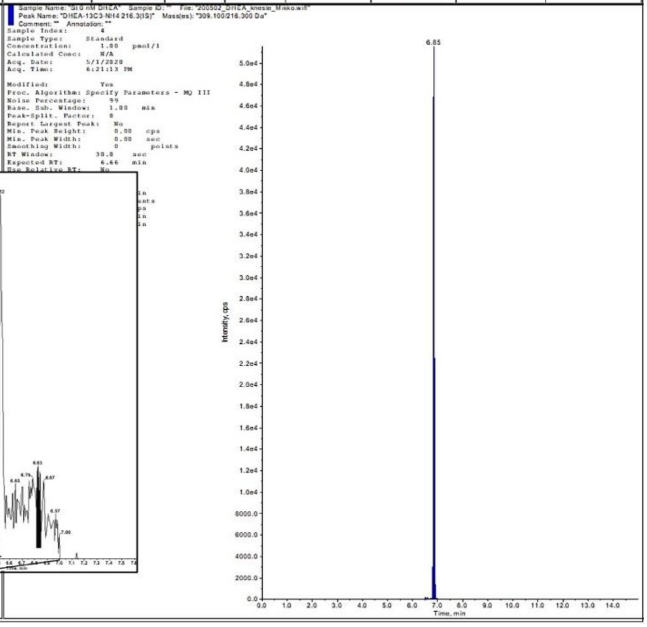

| | Sample Name | Sample Type | Analyte Peak Name | Analyte Peak Area (counts) | Analyte Peak Height (cps) | Analyte Concen tration (nmol/l) | Analyte Retention Time (min) | Analyte Signal To Noise | Area Ratio | IS Peak Name | IS Peak Area (counts) | IS Peak Height (cps) | IS Retention Time (min) | Calculated Concentrat ion (nmol/l) |
|---|---|---|---|---|---|---|---|---|---|---|---|---|---|---|
| 17 | 2 10956-2 | Unknown | DHEA-NH4 253.1 | 9.14e+002 | 9.89e+002 | N/A | 6.85 | N/A | -12.4 | DHEA- | -7.40e+00 | 2.60e+002 | 6.84 | < 0 |

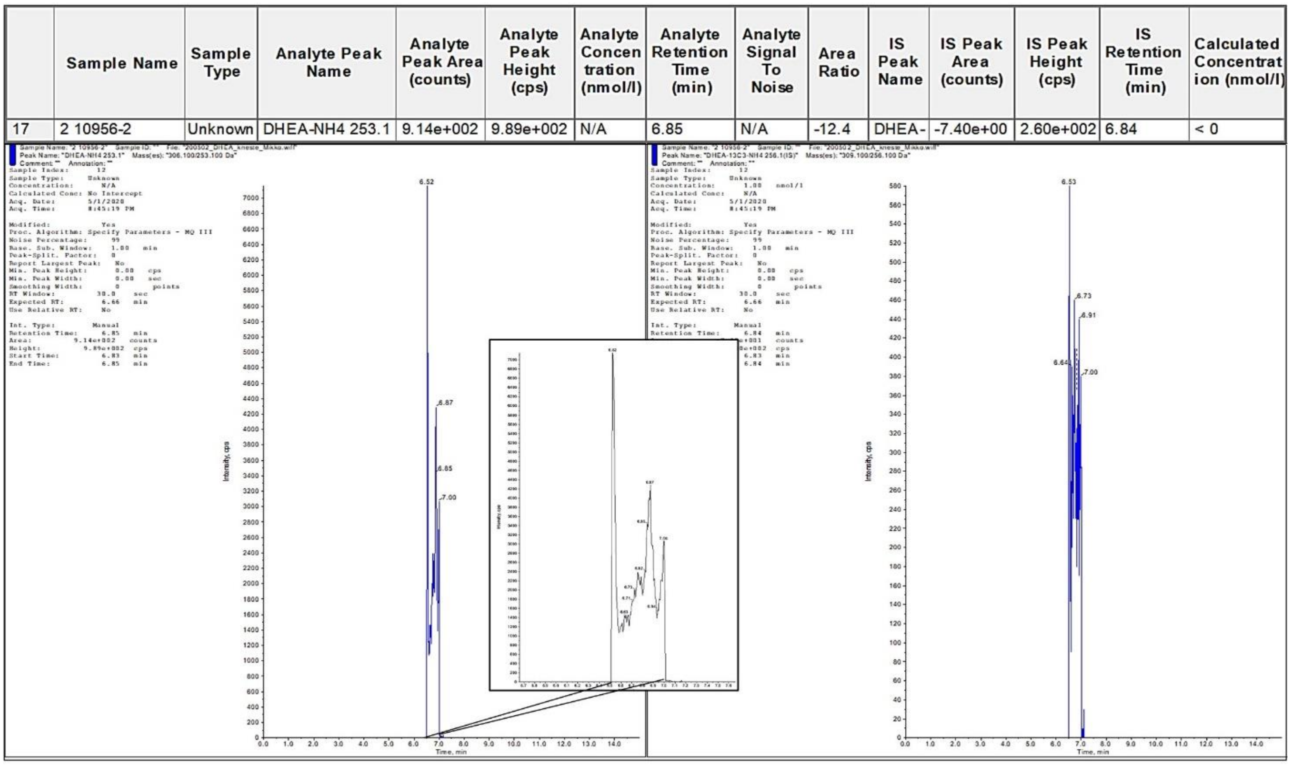

| | Sample Name | Sample Type | Analyte Peak Name | Analyte Peak Area (counts) | Analyte Peak Height (cps) | Analyte Concen tration (nmol/l) | Analyte Retention Time (min) | Analyte Signal To Noise | Area Ratio | IS Peak Name | IS Peak Area (counts) | IS Peak Height (cps) | IS Retention Time (min) | Calculated Concentrat ion (nmol/l) |
|---|---|---|---|---|---|---|---|---|---|---|---|---|---|---|
| 18 | 2 10956-2 | Unknown | DHEA-NH4 213.3 | 2.20e+002 | 3.81e+002 | N/A | 6.85 | N/A | -3.04 | DHEA- | -7.23e+00 | 2.50e+002 | 6.84 | < 0 |

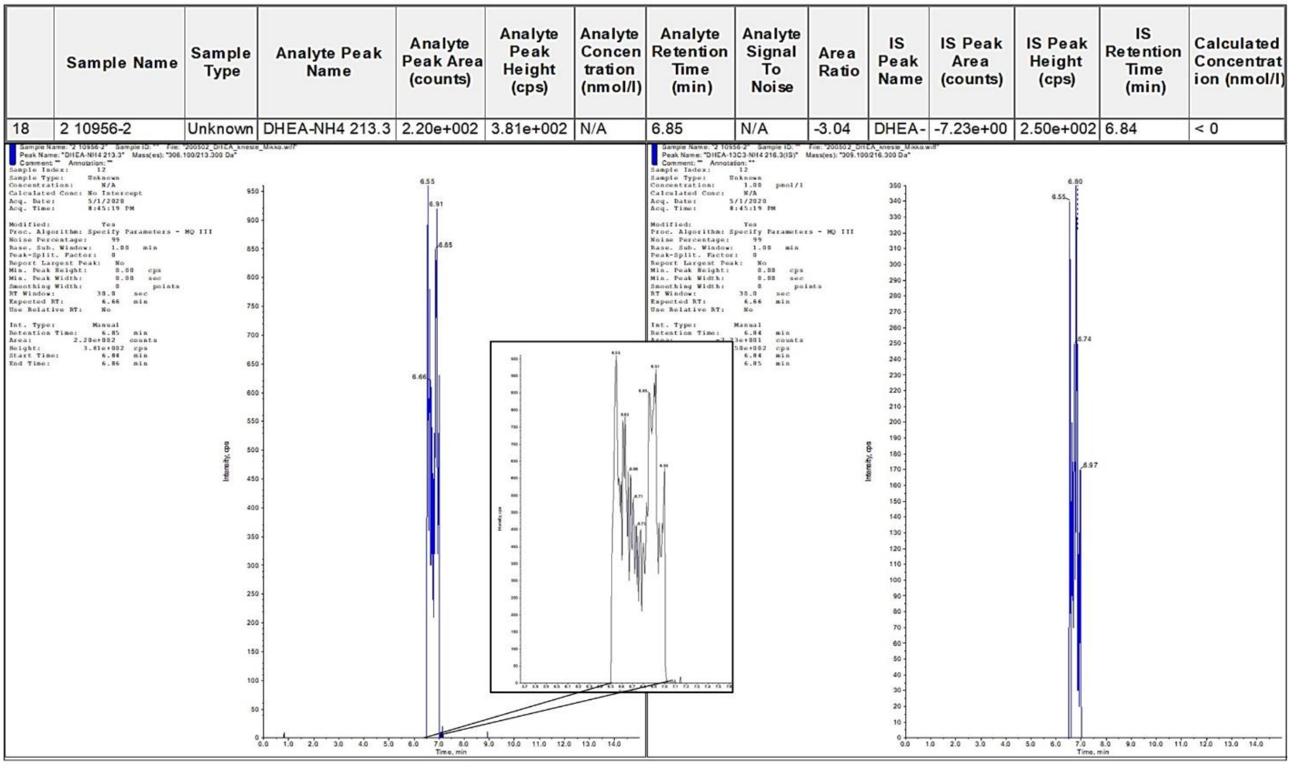

**Supplementary figure S2A: E1 LOQ chromatogram and S/N ratios.** Parent and transition ions $E_1$ [M−H]− m/z 269.1 to m/z 269.1 and m/z 145.0 in 10 pmol/L standard sample. Left panel shows calibrator chromatogram, while on the right is presented $^{13}$C-estrone IS. The high S/N ratio (36.5 and 26.6, respectively) permitted extrapolation of the LOQ to 5 pmol/L.

| | Sample Name | Sample Type | Analyte Peak Name | Analyte Peak Area (counts) | Analyte Peak Height (cps) | Analyte Concent ration (pmol/l) | Analyte Retention Time (min) | Analyte Signal To Noise | Area Ratio | IS Peak Name | IS Peak Area (counts) | IS Peak Height (cps) | IS Retention Time (min) | Calculated Concentration (pmol/l) |
|---|---|---|---|---|---|---|---|---|---|---|---|---|---|---|
| 4 | St 10 pM | Standard | E1 | 1.89e+003 | 6.89e+002 | 10.0 | 7.63 | 36.5 | 0.0149 | E1-13C3 | 1.27e+005 | 4.21e+004 | 7.64 | 10.1 |

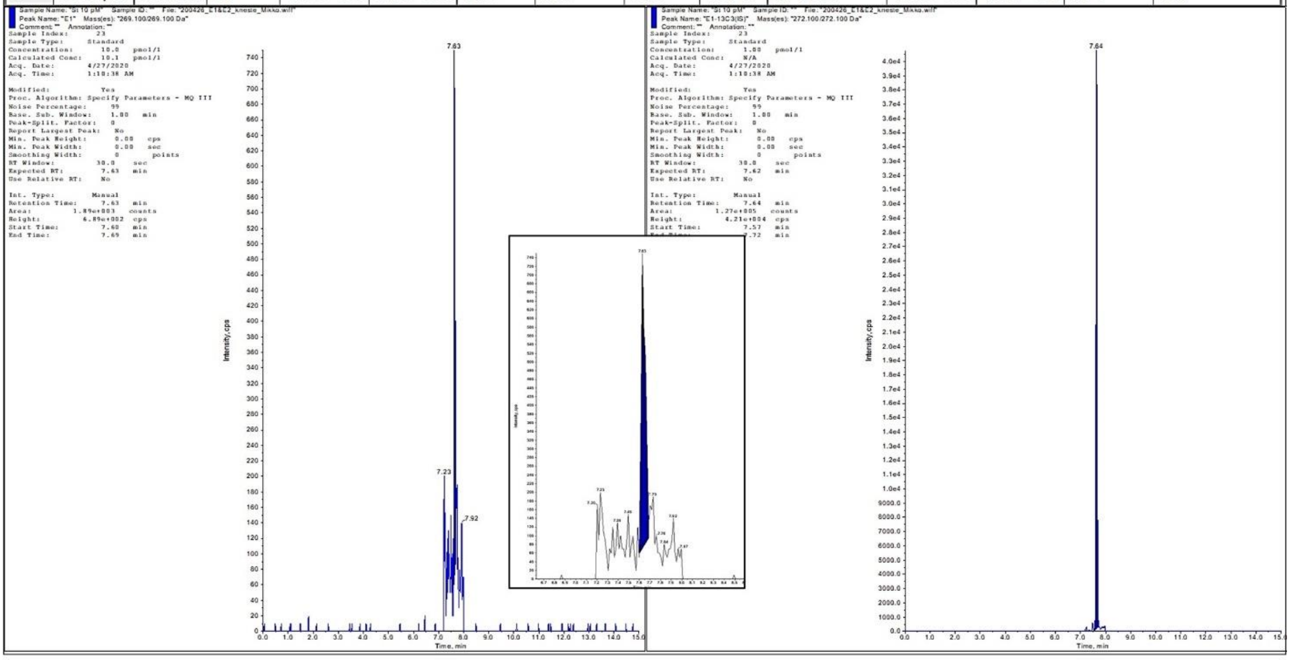

| | Sample Name | Sample Type | Analyte Peak Name | Analyte Peak Area (counts) | Analyte Peak Height (cps) | Analyte Concent ration (pmol/l) | Analyte Retention Time (min) | Analyte Signal To Noise | Area Ratio | IS Peak Name | IS Peak Area (counts) | IS Peak Height (cps) | IS Retention Time (min) | Calculated Concentration (pmol/l) |
|---|---|---|---|---|---|---|---|---|---|---|---|---|---|---|
| 3 | St 10 pM | Standard | E1 145.0 | 1.96e+004 | 5.99e+003 | 10.0 | 7.64 | 26.6 | 0.0197 | E1-13C3 148.0 | 9.98e+005 | 3.32e+005 | 7.64 | 10.1 |

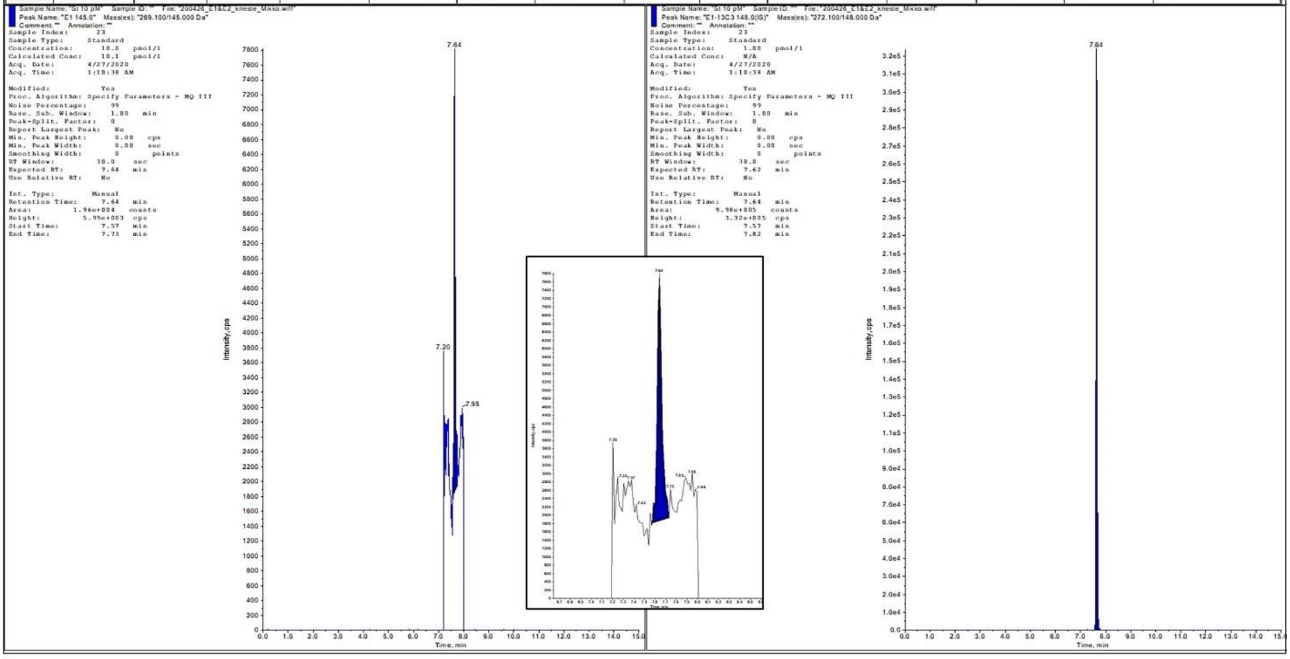

**Supplementary figure S2B: E2 LOQ chromatogram and S/N ratios.** Parent and transition ions $E_2$ [M−H]− m/z 271.2 to m/z 271.2 and m/z 183.1 in 10 pmol/L standard sample. Left panel shows calibrator chromatogram, while on the right is presented $^{13}$C-estradiol IS. The high S/N ratio permitted extrapolation of the LOQ to 5 pmol/L.

| | Sample Name | Sample Type | Analyte Peak Name | Analyte Peak Area (counts) | Analyte Peak Height (cps) | Analyte Concentration (pmol/l) | Analyte Retention Time (min) | Analyte Signal To Noise | Area Ratio | IS Peak Name | IS Peak Area (counts) | IS Peak Height (cps) | IS Retention Time (min) | Calculated Concentration (pmol/l) |
|---|---|---|---|---|---|---|---|---|---|---|---|---|---|---|
| 4 | St 10 pM | Standard | E2 | 4.69e+003 | 1.57e+003 | 10.0 | 7.50 | 69.4 | 0.00442 | E2-13C3 | 1.06e+006 | 3.34e+005 | 7.49 | 10.2 |

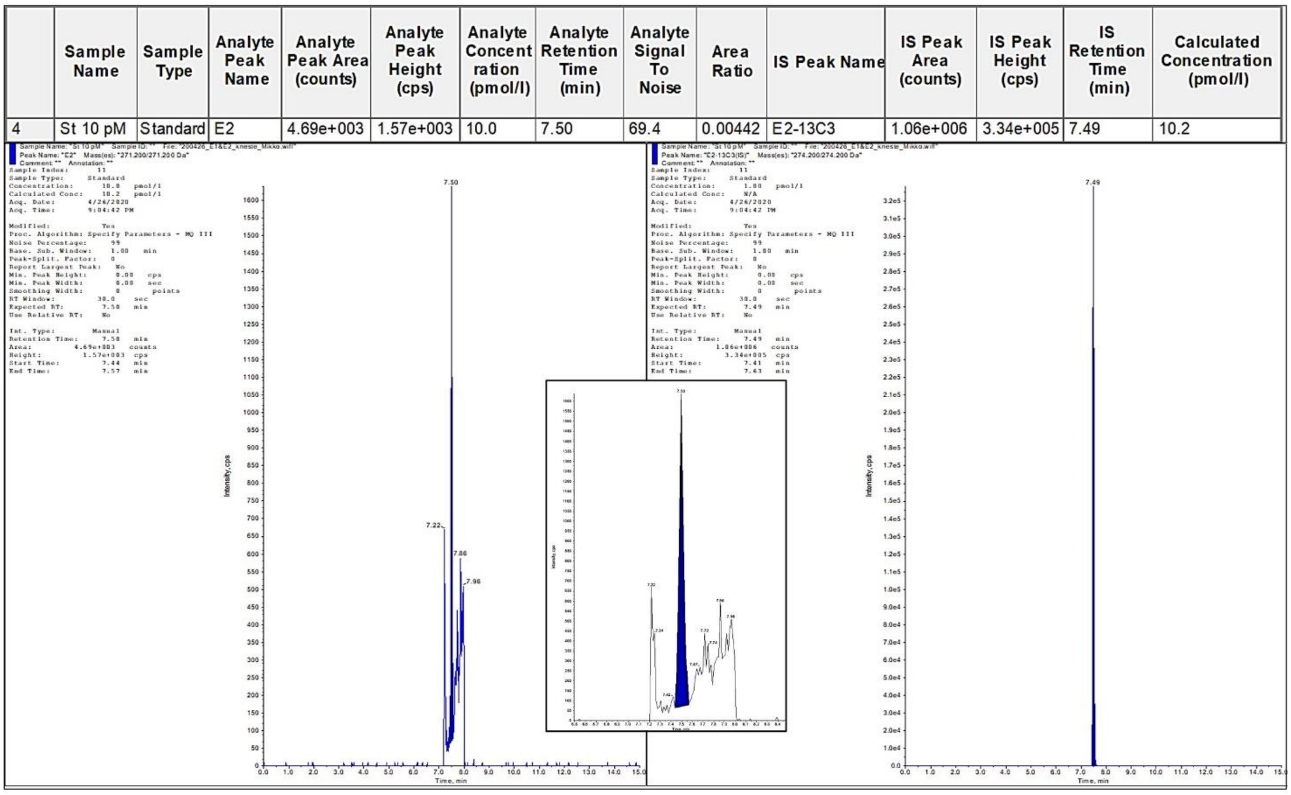

| | Sample Name | Sample Type | Analyte Peak Name | Analyte Peak Area (counts) | Analyte Peak Height (cps) | Analyte Concentration (pmol/l) | Analyte Retention Time (min) | Analyte Signal To Noise | Area Ratio | IS Peak Name | IS Peak Area (counts) | IS Peak Height (cps) | IS Retention Time (min) | Calculated Concentration (pmol/l) |
|---|---|---|---|---|---|---|---|---|---|---|---|---|---|---|
| 3 | St 10 pM | Standard | E2 183.1 | 3.32e+003 | 7.43e+002 | 10.0 | 7.50 | 15.2 | 0.00625 | E2-13C3 186.1 | 5.31e+005 | 1.64e+005 | 7.49 | 10.5 |

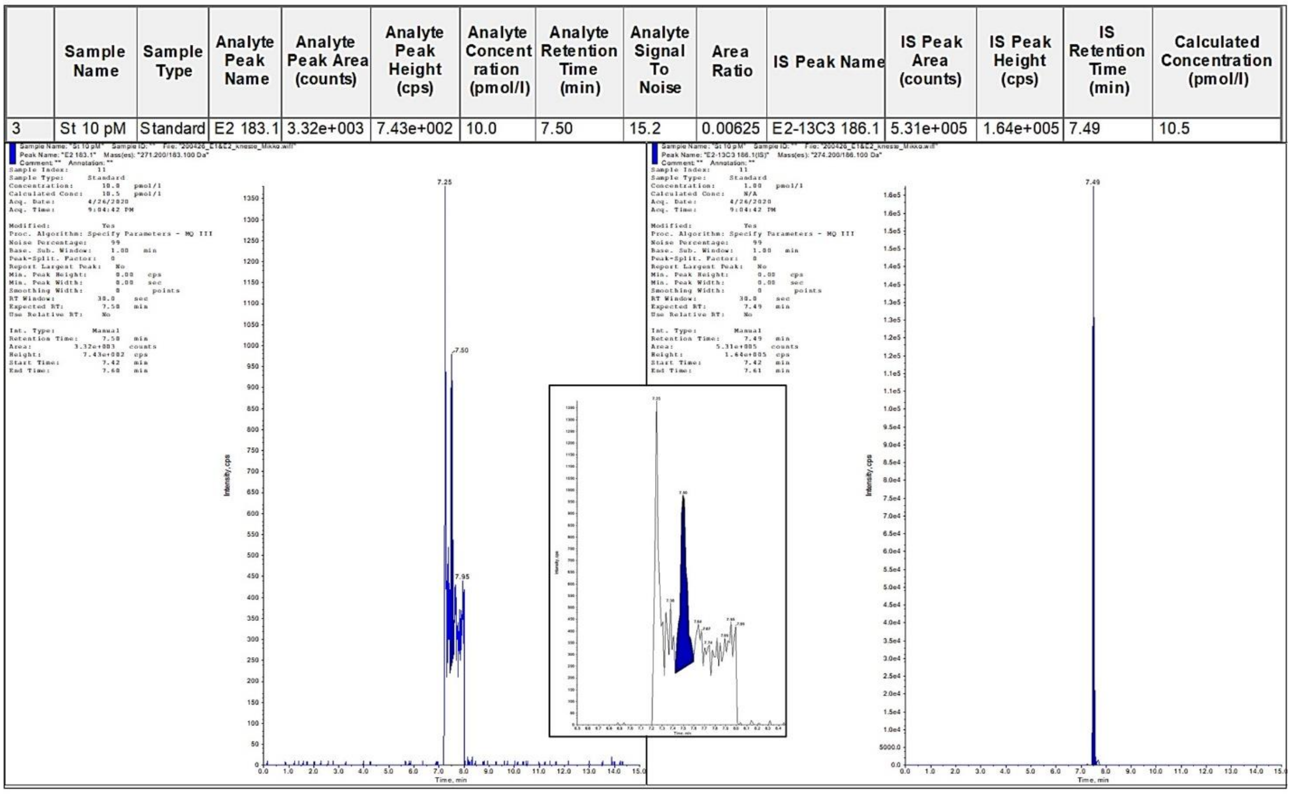

**Supplementary figure S2C: P4 LOQ chromatogram and S/N ratios.** Parent and transition ions P4 [M+H]+ m/z 315.1 to m/z 109.1 and m/z 96.9 in 50 pmol/L standard sample (S/N=132). Left panel shows calibrator chromatogram, while on the right is presented $^{13}$C-progesterone IS. The high S/N ratio permitted extrapolation of the LOQ to 10 pmol/L.

| | Sample Name | Sample Type | Analyte Peak Name | Analyte Peak Area (counts) | Analyte Peak Height (cps) | Analyte Concentration (nmol/l) | Analyte Retention Time (min) | Analyte Signal To Noise | Area Ratio | IS Peak Name | IS Peak Area (counts) | IS Peak Height (cps) | IS Retention Time (min) | Calculated Concentration (nmol/l) |
|---|---|---|---|---|---|---|---|---|---|---|---|---|---|---|
| 3 | St 0.05 nM Prog | Standard | PROG 96.9 | 2.01e+004 | 5.88e+003 | 0.0500 | 11.6 | 132. | 0.00291 | PROG-13C3 | 6.90e+006 | 2.15e+006 | 11.6 | 0.0512 |

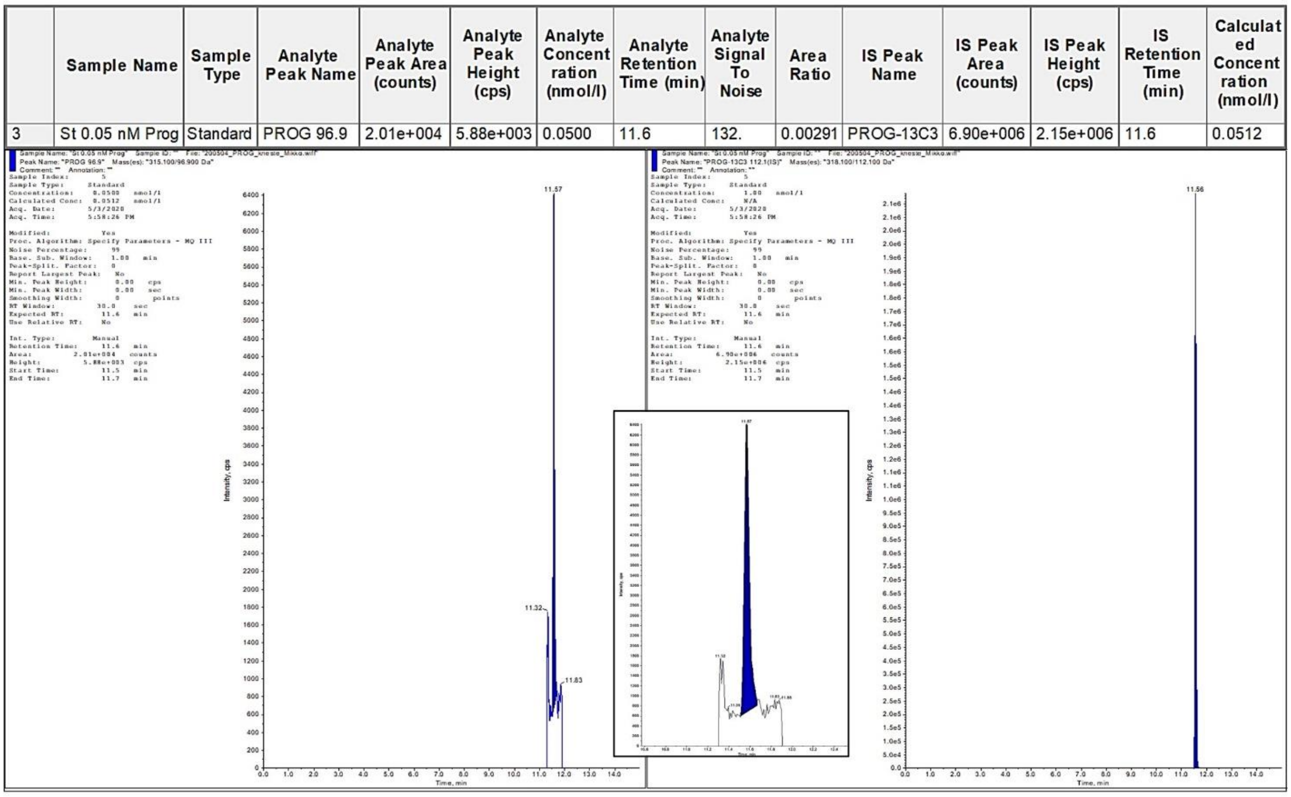


| | Sample Name | Sample Type | Analyte Peak Name | Analyte Peak Area (counts) | Analyte Peak Height (cps) | Analyte Concentration (nmol/l) | Analyte Retention Time (min) | Analyte Signal To Noise | Area Ratio | IS Peak Name | IS Peak Area (counts) | IS Peak Height (cps) | IS Retention Time (min) | Calculated Concentration (nmol/l) |
|---|---|---|---|---|---|---|---|---|---|---|---|---|---|---|
| 4 | St 0.05 nM Prog | Standard | PROG 109.1 | 2.38e+004 | 7.17e+003 | 0.0500 | 11.6 | 76.0 | 0.00345 | PROG-13C3 | 6.90e+006 | 2.15e+006 | 11.6 | 0.0495 |

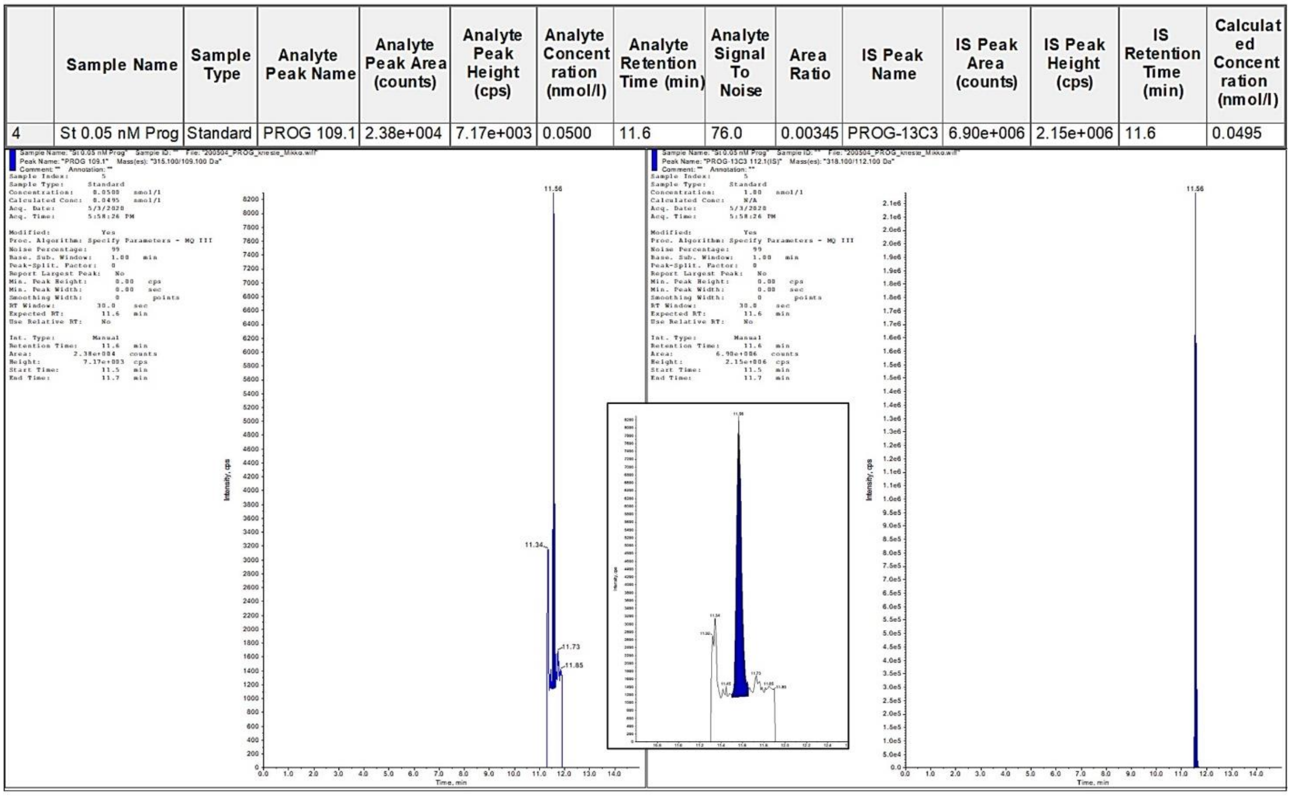


**Supplementary figure S2D: T4 LOQ chromatogram and S/N ratios.** Parent and transition ions T4 [M+H]+ m/z 289.1 to m/z 109.1 and m/z 97.0 in 50 pmol/L standard sample (S/N=99.5). Left panel shows calibrator chromatogram, while on the right is presented $^{13}$C-testosterone IS. The high S/N ratio permitted extrapolation of the LOQ to 10 pmol/L.

| | Sample Name | Sample Type | Analyte Peak Name | Analyte Peak Area (counts) | Analyte Peak Height (cps) | Analyte Concentration (nmol/l) | Analyte Retention Time (min) | Analyte Signal To Noise | Area Ratio | IS Peak Name | IS Peak Area (counts) | IS Peak Height (cps) | IS Retention Time (min) | Calculated Concentration (nmol/l) |
|---|---|---|---|---|---|---|---|---|---|---|---|---|---|---|
| 5 | St 0.05 nM | Standard | TESTO 97.0 | 3.71e+004 | 1.07e+004 | 0.0500 | 10.8 | 99.5 | 0.0130 | TESTO-13C3 100 | 2.84e+006 | 9.02e+005 | 10.8 | 0.0504 |

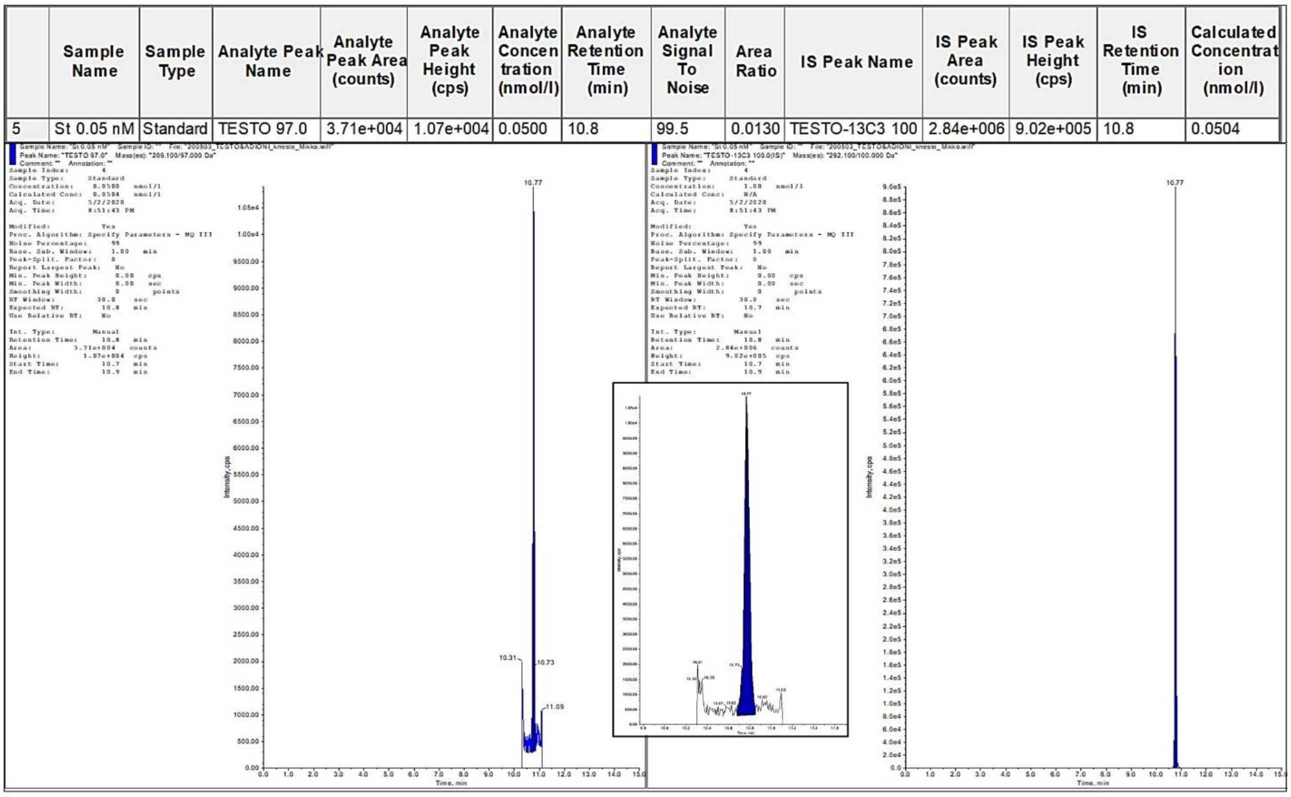


| | Sample Name | Sample Type | Analyte Peak Name | Analyte Peak Area (counts) | Analyte Peak Height (cps) | Analyte Concentration (nmol/l) | Analyte Retention Time (min) | Analyte Signal To Noise | Area Ratio | IS Peak Name | IS Peak Area (counts) | IS Peak Height (cps) | IS Retention Time (min) | Calculated Concentration (nmol/l) |
|---|---|---|---|---|---|---|---|---|---|---|---|---|---|---|
| 6 | St 0.05 nM | Standard | TESTO 109.1 | 3.47e+004 | 1.07e+004 | 0.0500 | 10.8 | 89.8 | 0.0134 | TESTO-13C3 112 | 2.58e+006 | 7.98e+005 | 10.8 | 0.0503 |

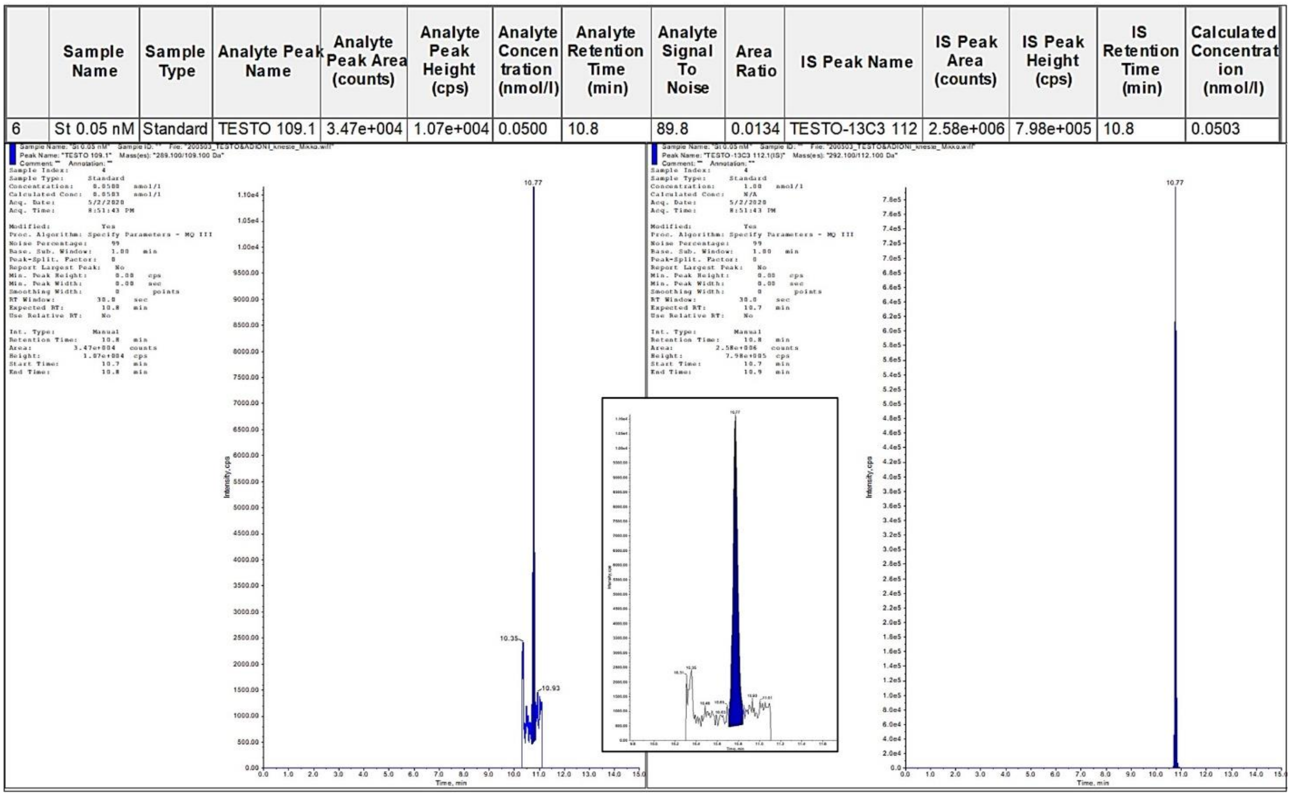


**Supplementary figure S2E: A4 LOQ chromatogram and S/N ratios.** Parent and transition ions A4 [M+H]+ m/z 287.1 to m/z 109.2 and m/z 97.2 in 50 pmol/L standard sample (S/N=171.0). Left panel shows calibrator chromatogram, while on the right is presented $^{13}$C-androstenedione IS. The high S/N ratio permitted extrapolation of the LOQ to 10 pmol/L.

| | Sample Name | Sample Type | Analyte Peak Name | Analyte Peak Area (counts) | Analyte Peak Height (cps) | Analyte Concentration (nmol/l) | Analyte Retention Time (min) | Analyte Signal To Noise | Area Ratio | IS Peak Name | IS Peak Area (counts) | IS Peak Height (cps) | IS Retention Time (min) | Calculated Concentration (nmol/l) |
|---|---|---|---|---|---|---|---|---|---|---|---|---|---|---|
| 8 | St 0.05 nM | Standard | ADIONI 109. | 7.27e+004 | 2.20e+004 | 0.0500 | 10.7 | 171. | 0.0374 | ADIONI-13C3 11 | 1.94e+006 | 5.91e+005 | 10.7 | 0.0494 |

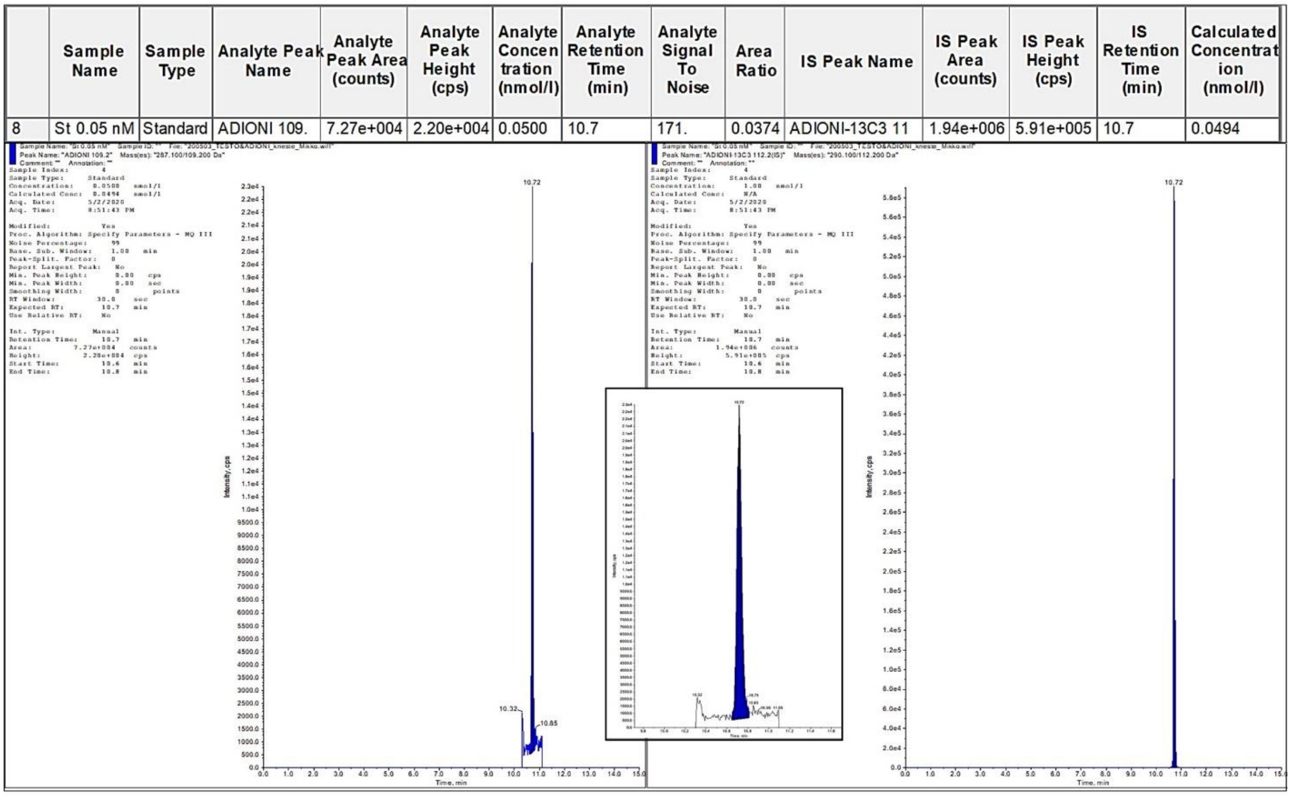

| | Sample Name | Sample Type | Analyte Peak Name | Analyte Peak Area (counts) | Analyte Peak Height (cps) | Analyte Concentration (nmol/l) | Analyte Retention Time (min) | Analyte Signal To Noise | Area Ratio | IS Peak Name | IS Peak Area (counts) | IS Peak Height (cps) | IS Retention Time (min) | Calculated Concentration (nmol/l) |
|---|---|---|---|---|---|---|---|---|---|---|---|---|---|---|
| 7 | St 0.05 nM | Standard | ADIONI 97.2 | 9.95e+004 | 3.05e+004 | 0.0500 | 10.7 | 76.4 | 0.0375 | ADIONI-13C3 10 | 2.65e+006 | 8.14e+005 | 10.7 | 0.0496 |

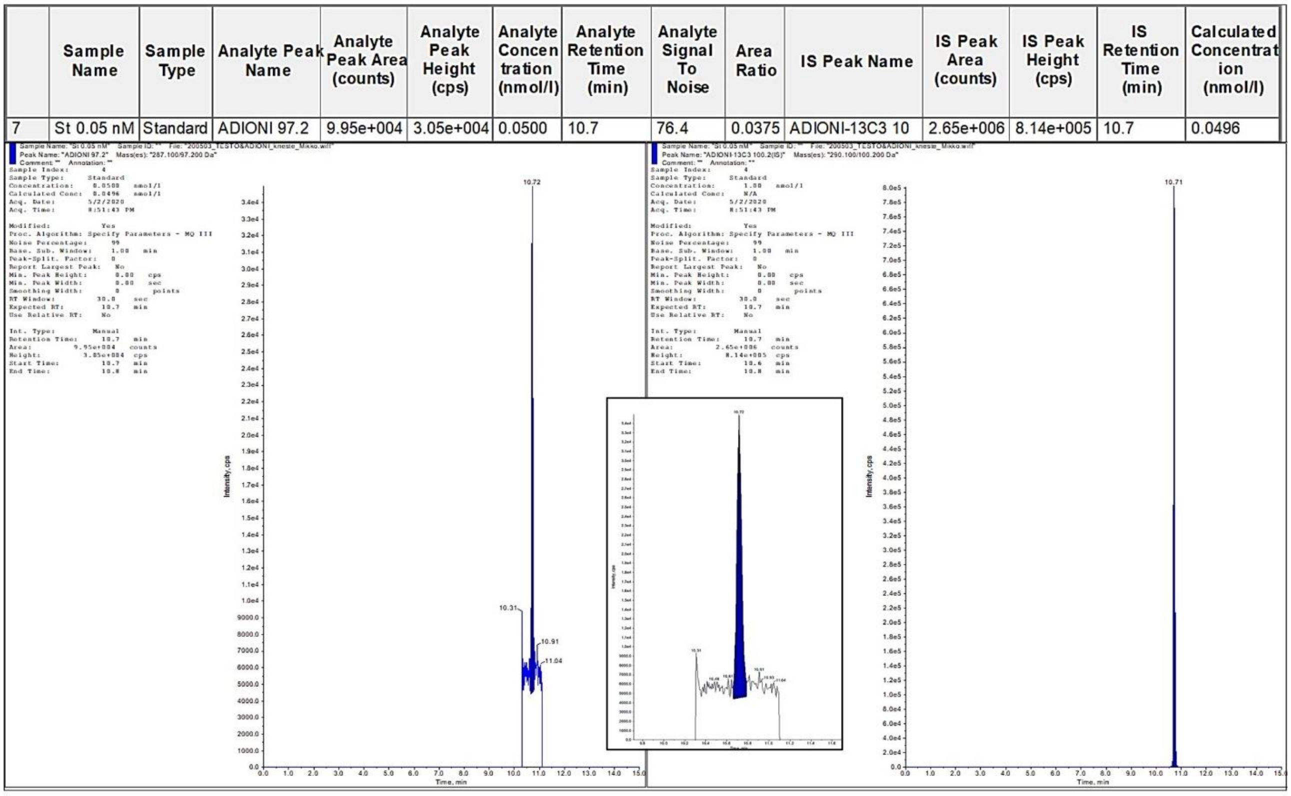

**Supplementary figure S2F: DHEA-LOQ chromatogram and S/N ratios.** Parent and transition ions – ammonium adduct [M+NH4]+ m/z 306.1 to m/z 253.1 and m/z 213.3 in 0.5 nmol/L standard sample (S/N=182.0). Left panel shows calibrator chromatogram, while on the right is presented $^{13}$C-DHEA IS. The high S/N ratio permitted extrapolation of the LOQ to 0.1 nmol/L.

| | Sample Name | Sample Type | Analyte Peak Name | Analyte Peak Area (counts) | Analyte Peak Height (cps) | Analyte Concentration (nmol/l) | Analyte Retention Time (min) | Analyte Signal To Noise | Area Ratio | IS Peak Name | IS Peak Area (counts) | IS Peak Height (cps) | IS Retention Time (min) | Calculated Concentration (nmol/l) |
|---|---|---|---|---|---|---|---|---|---|---|---|---|---|---|
| 7 | St 0.5 nM DHEA | Standard | DHEA-NH4 253.1 | 4.29e+004 | 1.71e+004 | 0.500 | 6.86 | 182. | 0.0346 | DHEA- | 1.24e+006 | 4.66e+005 | 6.86 | 0.498 |

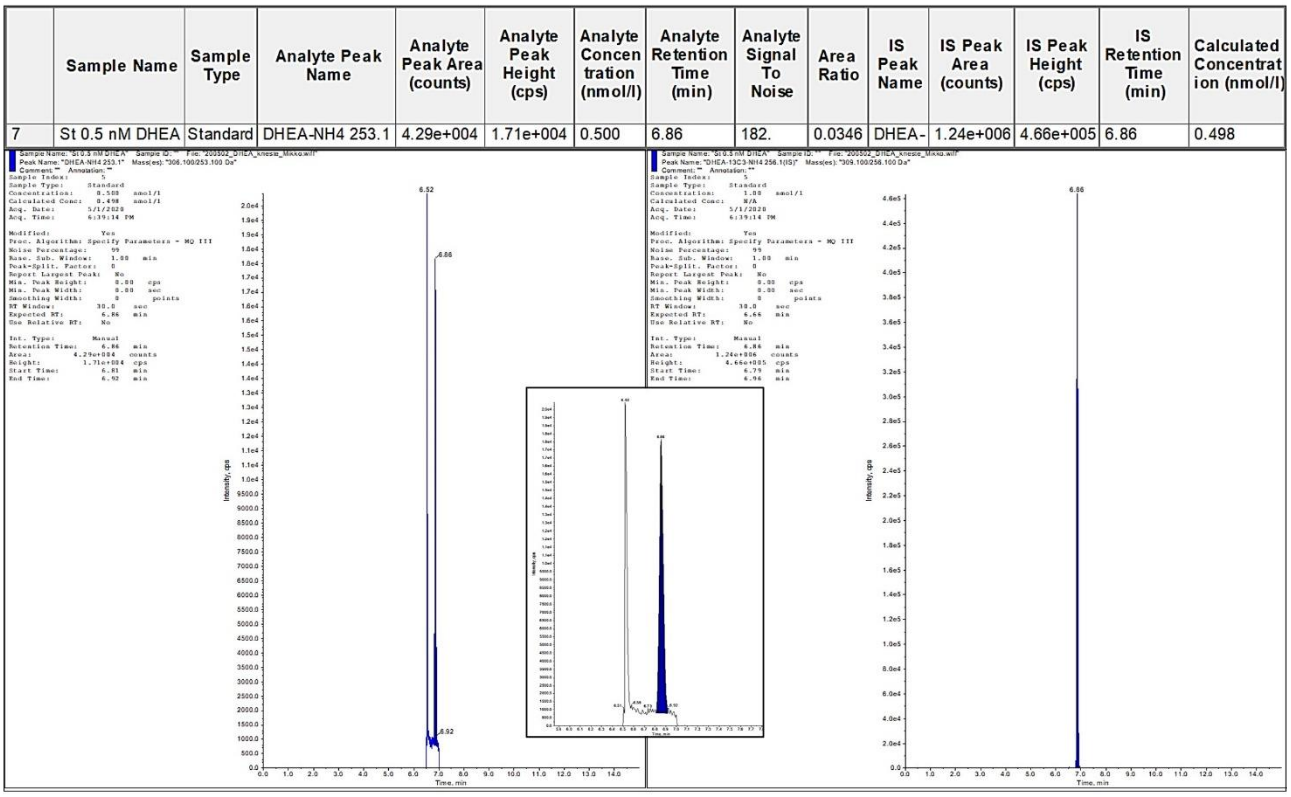


| | Sample Name | Sample Type | Analyte Peak Name | Analyte Peak Area (counts) | Analyte Peak Height (cps) | Analyte Concentration (nmol/l) | Analyte Retention Time (min) | Analyte Signal To Noise | Area Ratio | IS Peak Name | IS Peak Area (counts) | IS Peak Height (cps) | IS Retention Time (min) | Calculated Concentration (nmol/l) |
|---|---|---|---|---|---|---|---|---|---|---|---|---|---|---|
| 8 | St 0.5 nM DHEA | Standard | DHEA-NH4 213.3 | 6.31e+003 | 2.45e+003 | 0.500 | 6.86 | 56.5 | 0.0433 | DHEA- | 1.46e+005 | 5.39e+004 | 6.85 | 0.512 |

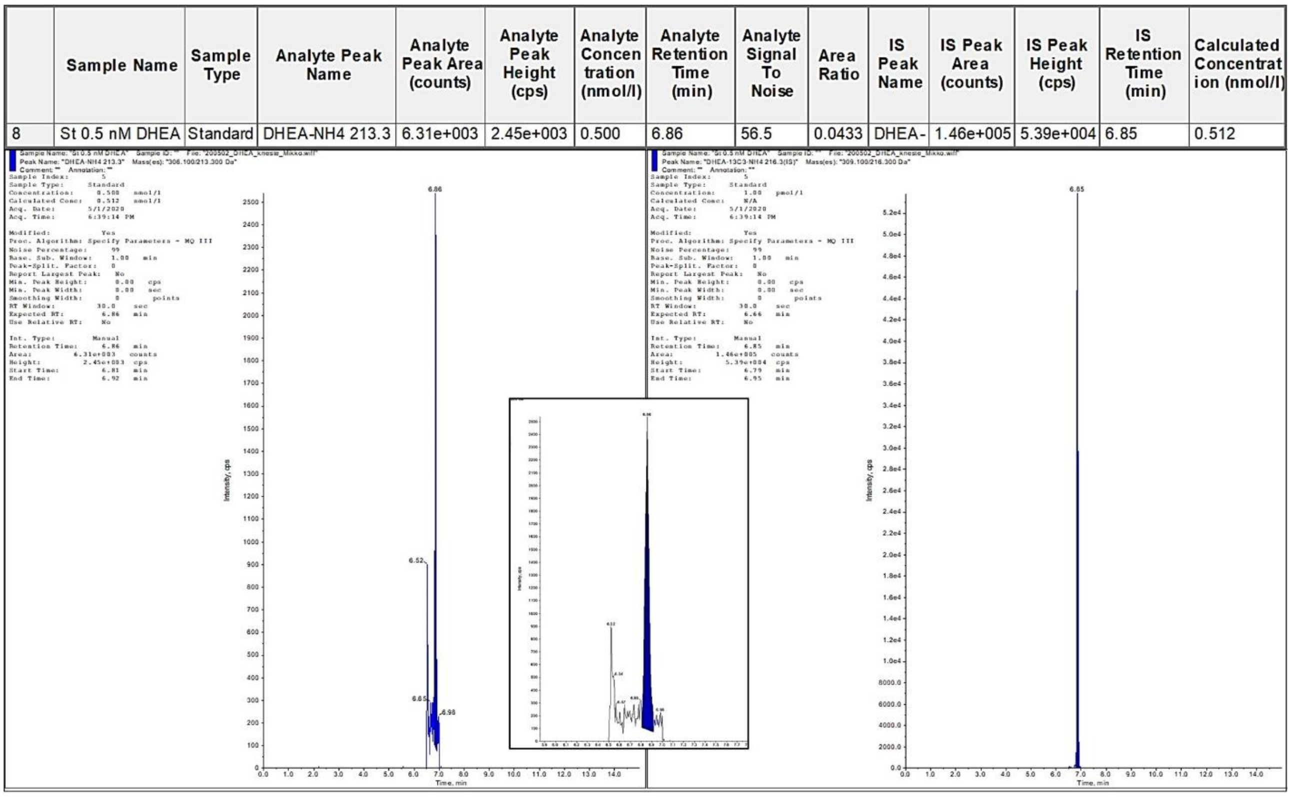


**Supplementary figure S3: T4 .** Parent and transition T4 [M+H]+ m/z 289.1 to m/z 109.1 and m/z 97.0. Left panel shows analyte chromatogram, while on the right $^{13}$C-testosterone IS.

| a | Sample Name | Sample Type | Analyte Peak Name | Analyte Peak Area (counts) | Analyte Peak Height (cps) | Analyte Concentration (nmol/l) | Analyte Retention Time (min) | Analyte Signal To Noise | Area Ratio | IS Peak Name | IS Peak Area (counts) | IS Peak Height (cps) | IS Retention Time (min) | Calculated Concentration (nmol/l) |
|---|---|---|---|---|---|---|---|---|---|---|---|---|---|---|
| 85 | 15 10956-15 | Unknown | TESTO 97.0 | 3.67e+005 | 1.10e+005 | N/A | 10.8 | 954. | 0.0472 | TESTO-13C3 | 7.77e+006 | 2.48e+006 | 10.8 | 0.801 |

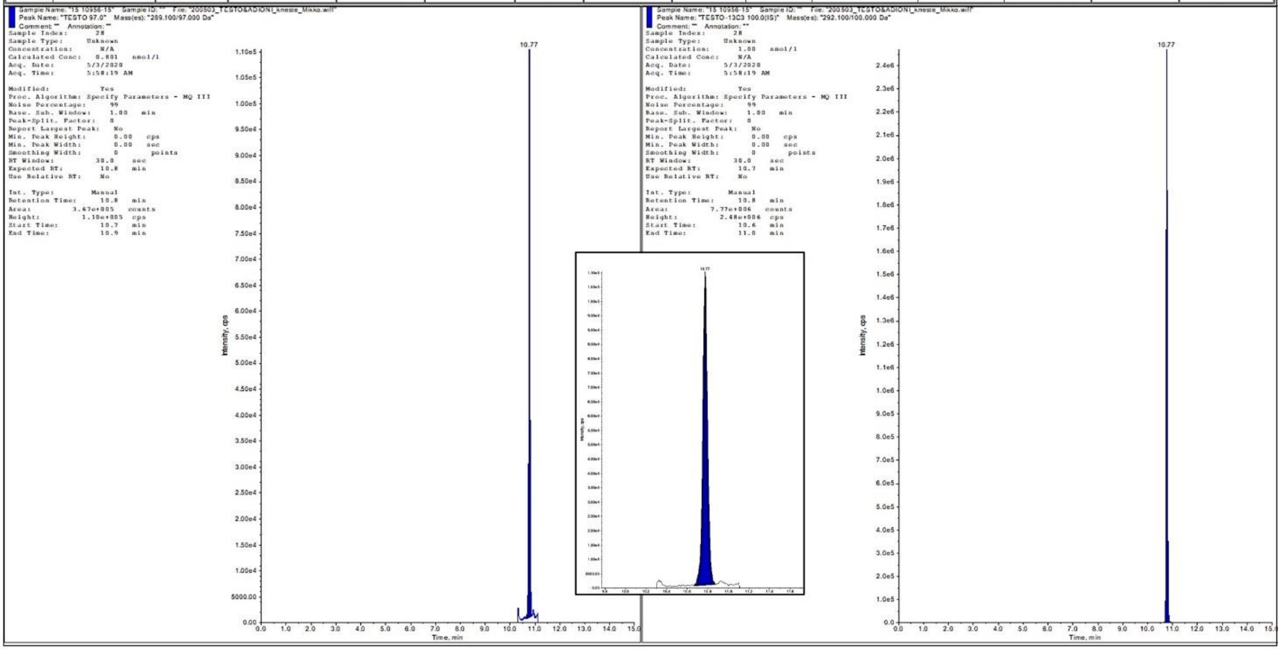

| | Sample Name | Sample Type | Analyte Peak Name | Analyte Peak Area (counts) | Analyte Peak Height (cps) | Analyte Concentration (nmol/l) | Analyte Retention Time (min) | Analyte Signal To Noise | Area Ratio | IS Peak Name | IS Peak Area (counts) | IS Peak Height (cps) | IS Retention Time (min) | Calculated Concentration (nmol/l) |
|---|---|---|---|---|---|---|---|---|---|---|---|---|---|---|
| 86 | 15 10956-15 | Unknown | TESTO 109.1 | 3.48e+005 | 1.10e+005 | N/A | 10.8 | 405. | 0.0491 | TESTO-13C3 | 7.07e+006 | 2.29e+006 | 10.8 | 0.833 |

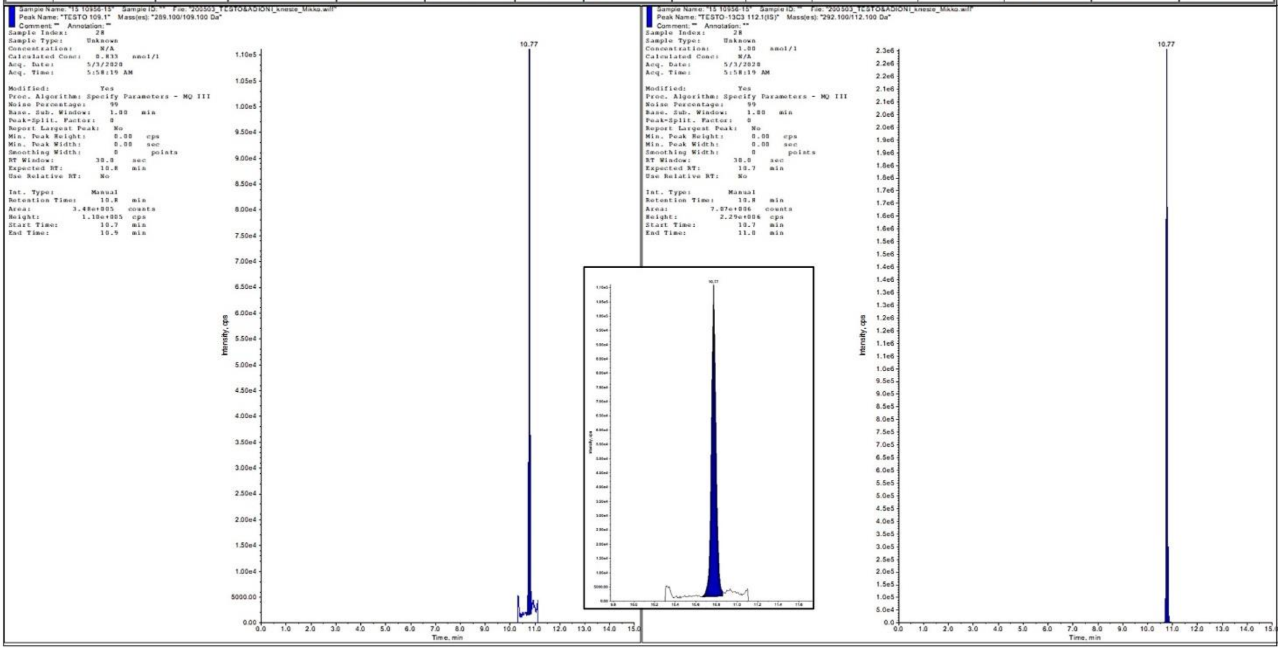

| b | Sample Name | Sample Type | Analyte Peak Name | Analyte Peak Area (counts) | Analyte Peak Height (cps) | Analyte Concentration (nmol/l) | Analyte Retention Time (min) | Analyte Signal To Noise | Area Ratio | IS Peak Name | IS Peak Area (counts) | IS Peak Height (cps) | IS Retention Time (min) | Calculated Concentration (nmol/l) |
|---|---|---|---|---|---|---|---|---|---|---|---|---|---|---|
| 53 | 7 10956-7 | Unknown | TESTO 97.0 | 2.45e+003 | 1.09e+003 | N/A | 10.8 | N/A | 0.00022 | TESTO-13C3 | 1.08e+007 | 3.36e+006 | 10.8 | 0.00188 |

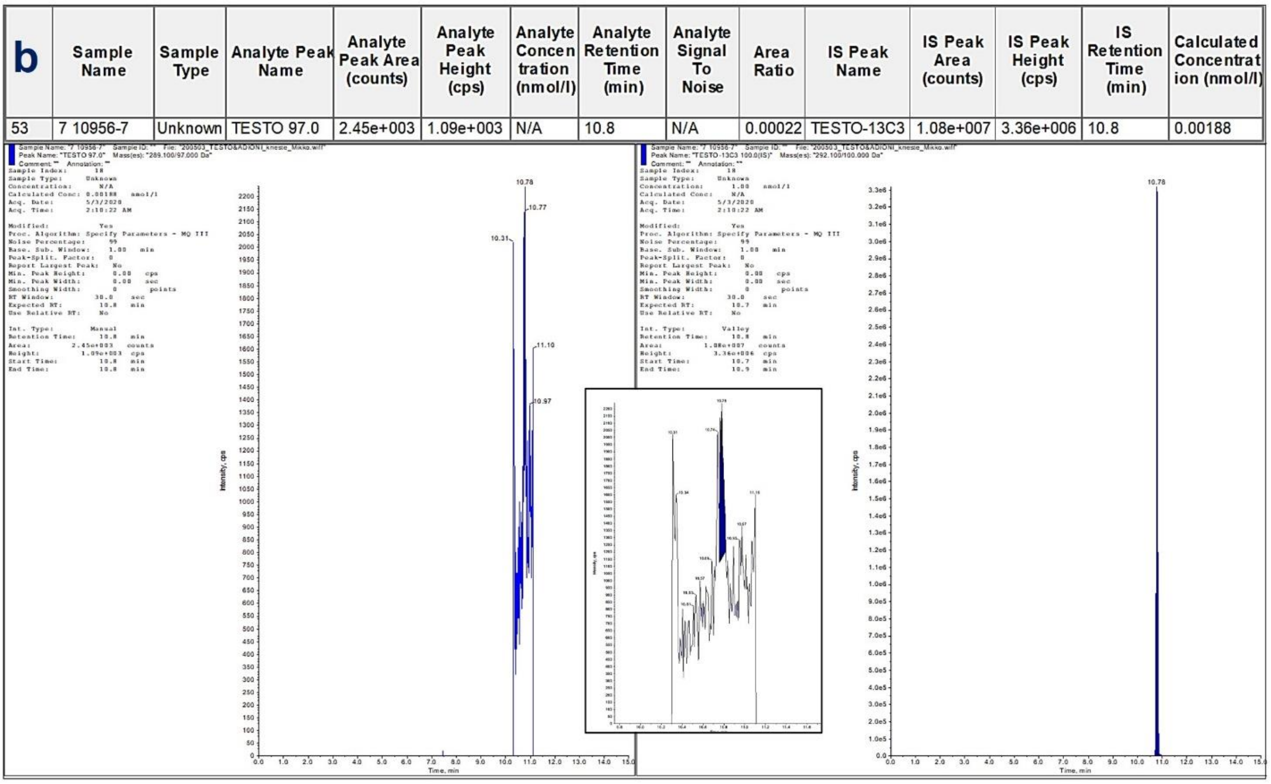

| | Sample Name | Sample Type | Analyte Peak Name | Analyte Peak Area (counts) | Analyte Peak Height (cps) | Analyte Concentration (nmol/l) | Analyte Retention Time (min) | Analyte Signal To Noise | Area Ratio | IS Peak Name | IS Peak Area (counts) | IS Peak Height (cps) | IS Retention Time (min) | Calculated Concentration (nmol/l) |
|---|---|---|---|---|---|---|---|---|---|---|---|---|---|---|
| 54 | 7 10956-7 | Unknown | TESTO 109.1 | 3.03e+003 | 1.42e+003 | N/A | 10.8 | N/A | 0.00030 | TESTO-13C3 | 9.91e+006 | 3.01e+006 | 10.8 | 0.00168 |

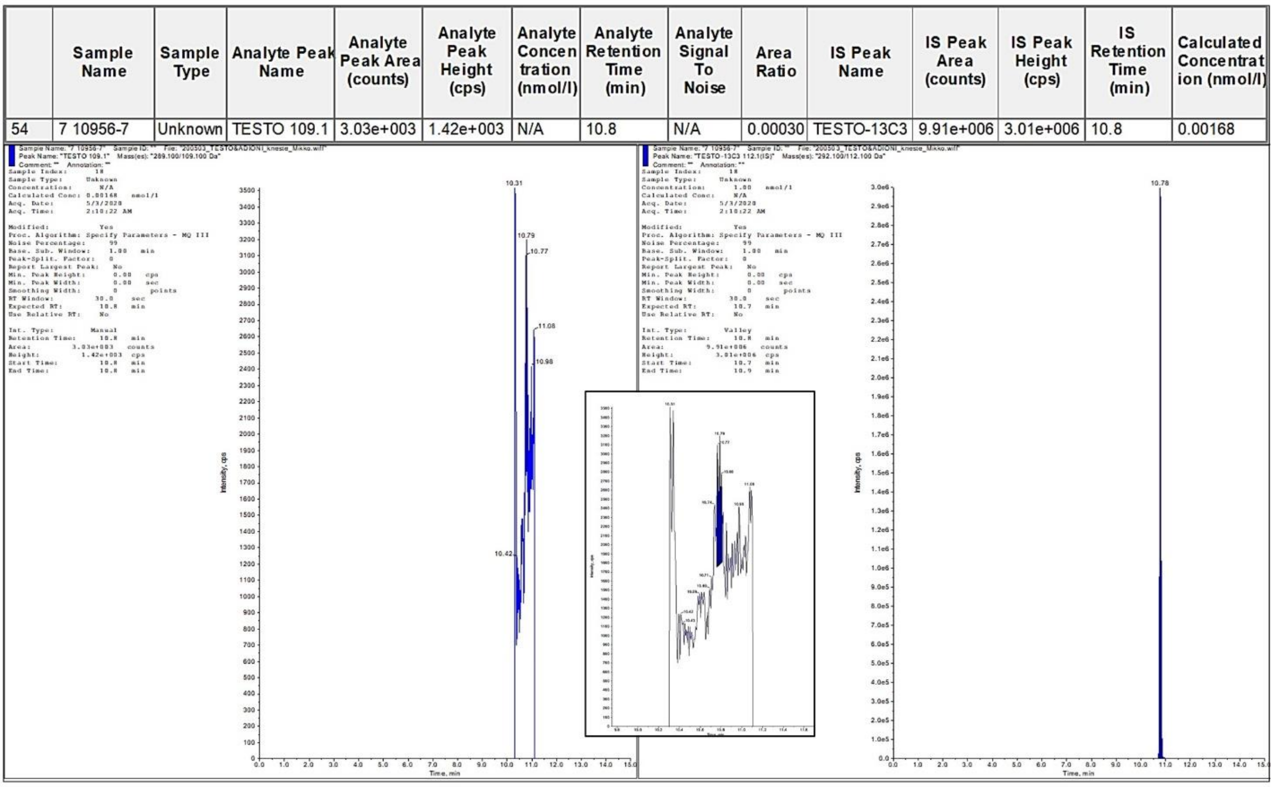

Supplement: Supplementary file 1 [file ijms-23-14864-s001.zip › ijms-2024599-supplementary.pdf]
